# Shear modulus data for the human lens determined from a spinning lens test

**DOI:** 10.1016/j.exer.2012.01.011

**Published:** 2012-04

**Authors:** G.S. Wilde, H.J. Burd, S.J. Judge

**Affiliations:** a Department of Engineering Science, University of Oxford, Parks Road, Oxford OX1 3PJ, UK; b Department of Physiology, Anatomy and Genetics, University of Oxford, OX1 3QX, UK

**Keywords:** human lens, stiffness, ageing, shear modulus

## Abstract

The paper describes a program of mechanical testing on donated human eye bank lenses. The principal purpose of the tests was to obtain experimental data on the shear modulus of the lens for use in future computational models of the accommodation process. Testing was conducted using a procedure in which deformations are induced in the lens by spinning it about its polar axis. Shear modulus data were inferred from these observed deformations by means of a finite element inverse analysis procedure in which the spatial variation of the shear modulus within the lens is represented by an appropriate function (see Burd et al., 2011 for a detailed specification of the design of the spinning lens test rig, experimental protocols and associated data analysis procedures that were employed in the tests). Inferred data on lens shear modulus are presented for a set of twenty-nine lenses in the age range 12 years to 58 years. The lenses were tested between 47 h and 110 h from the time of death (average *post-mortem* time 74 h). Care was taken to exclude any lenses that had been affected by excessive post-mortem swelling, or any lenses that had suffered mechanical damage during storage, transit or the testing process. The experimental data on shear modulus indicate that, for young lenses, the *cortex* is stiffer than the nucleus. The shear modulus of the nucleus and cortex both increase with increasing age. The shear modulus of the nucleus increases more rapidly than the cortex with the consequence that from an age of about 45 years onwards the *nucleus* is stiffer than the cortex. The principal shear modulus data presented in the paper were obtained by testing at a rotational speed of 1000 rpm. Supplementary tests were conducted at rotational speeds of 700 rpm and 1400 rpm. The results from these supplementary tests are in good agreement with the data obtained from the principal 1000 rpm tests. Studies on the possible effects of lens drying during the test suggested that this factor is unlikely to have led to significant errors in the experimental determination of the shear modulus. The shear modulus data presented in the paper are used to develop ‘age-stiffness’ models to represent the shear modulus of the lens as a function of age. These models are in a form that may be readily incorporated in a finite element model of the accommodation process. A comparison is attempted between the shear modulus data presented in the current paper and equivalent data published by previous authors. This comparison highlights various limitations and inconsistencies in the data sets.

## Highlights

► A set of shear modulus data have been obtained for the human lens. ► Data were obtained using a spinning lens test. ► The results show that the nucleus stiffens more rapidly with age than the cortex. ► The data are in a form that can be readily employed in a finite element model of accommodation.

## 1 Introduction

### 1.1 Purpose

Accommodation of the human eye is achieved by means of the deformations that are induced in the lens in response to changes in radial force applied to the lens equator, via the zonules, when the ciliary body contracts (e.g. von Helmholtz, 1855).

These deformations influence the optical performance of the eye in two distinct ways. Firstly, a discontinuity in refractive index exists at the interface between the anterior surface of the lens and the aqueous. Light rays are refracted at this interface and, as a consequence, the increase in anterior lens curvature that occurs during accommodation contributes to an increase in the overall optical power of the eye. The discontinuity of refractive index at the interface between the posterior surface and the vitreous provides a similar component of the accommodation mechanism. Separately, it is widely understood that the refractive index of the lens varies spatially within it (e.g. Jones et al., 2005). It seems possible that the internal deformations developed in the lens during accommodation will act to distort this internal refractive index distribution leading to a further lenticular contribution to the accommodation mechanism (e.g. Navarro et al., 2007).

It is clear that the optical performance of the eye during the accommodation process depends on the mechanical performance of the lens. Studies on lens mechanics therefore form an important part of the continued development of an improved understanding of the accommodation performance of the human eye.

A variety of approaches have been adopted by previous authors to investigate the mechanical behaviour of the human lens during accommodation. One such approach is to apply computational modelling procedures, such as the finite element method, to construct mechanical models of the process (e.g. Burd et al., 2002; Hermans et al., 2006, 2008; Stachs et al., 2006; Weeber and van der Heijde, 2007). In this approach, a numerical model is constructed in which details of the geometric, kinematic and constitutive behaviour of the individual components of the accommodation apparatus are incorporated. (The term ‘constitutive behaviour’ in this context refers – in broad terms – to relationships between the stresses and strains within the relevant tissues). The success of these computational modelling procedures is strongly dependent on the use of an appropriate mathematical formulation (termed ‘constitutive model’) to represent the constitutive behaviour of the various tissues that form the accommodation apparatus.

A constitutive model invariably requires the specification of one or more material parameters to calibrate the model for a particular material or tissue of interest. These parameters are generally determined by appropriate experimentation on the relevant material or tissue. Reliable data on these material parameters are a prerequisite of any satisfactory model.

The current paper presents experimental data on one material parameter – the shear modulus of the lens substance – that is a key ingredient of any mechanical model of accommodation in which an elastic constitutive model is employed for the lens substance. These data have been obtained by measurements on fresh donated human lenses. Appropriate ethical permissions were sought, and obtained, prior to the start of the tests. In a previous paper (Burd et al., 2011) we provide a specification of the spinning lens rig together with the protocol that was used to conduct these experiments and process the data. To emphasise the close relationship between the shear modulus data presented in the current paper and the methods described in Burd et al. (2011), we refer below to Burd et al. (2011) as the ‘methods paper’.

### 1.2 Previous data on human lens stiffness

Relatively little published experimental data currently exist on the stiffness of the human lens substance. Considering the fundamental importance of the accommodation process for day-to-day life, and the widespread current interest in the possibility of developing surgical or medical procedures to slow down or reverse the effects of presbyopia, it is remarkable that published experimental data on the mechanical properties of the human lens are so limited.

The first published experiment to measure the stiffness of the human lens, in which the data are presented in terms of a stiffness parameter that can be used to calibrate a conventional constitutive model, was described by Fisher (1971). In this experiment, excised lenses were placed on a rotor and the shape changes induced in the lens surface by spinning it about its polar axis were determined by flash photography. Separate estimates of the Young's modulus of the nucleus and cortex were then made on the basis of these observed shape changes. The Fisher results remained a unique source of experimental data on human lens stiffness until the publication by Heys et al. (2004) of shear modulus data determined by micro-indentation testing. In subsequent years, data on dynamic measurements on a sample comprising lens fragments (Weeber et al., 2005) and shear modulus data from dynamic indentation testing (Weeber et al., 2007) have been published. Some data on lens Young's modulus obtained using an acoustic method are given by Hollman et al. (2007).

An analyst seeking to embed currently-published data on lens stiffness in a computational model of the accommodation process is currently faced with multiple difficulties. The Fisher (1971) Young's modulus data, for example, were determined from the images collected during the test on the basis of various simplifying assumptions. Detailed scrutiny of these data, and the modelling assumptions on which they are based (Burd et al., 2006), indicates that they suffer from a range of systematic errors; questions therefore exist on the reliability of this particular data set. The indentation test results of Heys et al. (2004) and Weeber et al. (2007) were conducted on an approximately equatorial plane formed by cutting the lens into two parts. The tests were conducted on previously frozen lenses and questions exist on the possible effect of the freezing process on the measured stiffness data. Although both data sets provide a strong indication of the spatial variation of stiffness within the lens, because the indentation was restricted to a single loading direction on a single plane there is no unique way of extending these data to provide a representation of stiffness of the lens as a whole as is required, for example, in a finite element model of the lens. (Although one possible approach to extending these data to form a spatial representation for the lens as a whole is given by Weeber and van der Heijde, 2007; Riehemann et al., 2011). The data of Weeber et al. (2005) relate to the dynamic behaviour of lens fragments; there is no obvious way of mapping these data onto a representation of the mechanics of the whole lens in a way that reflects any spatial non-homogeneities that may exist. The data of Hollman et al. (2007) provide values of Young's modulus determined at specific internal locations in a small number of lenses, but there are insufficient data to determine the spatial variation of stiffness for the lens as a whole or to provide much detail of the way in which the stiffness of the lens changes with age.

The shear modulus data presented in the current paper provide the basis for a spatial model of lens heterogeneity that does not rely on the use of the extrapolation of values determined from measurements made at discrete points within the lens. The data are presented in forms that are readily incorporated in finite element models of the accommodation process.

It is noticeable, when reviewing the literature, that few attempts have been made to compare, systematically, the numerical values of published data on lens stiffness from different published sources. (Although brief comparisons are given in Weeber et al. (2007) and Riehemann et al., 2011). Comparisons of this sort allow an assessment to be made of whether the data presented in the literature provide a consistent picture or whether inconsistencies and outliers exist. The absence of any detailed comparative studies of this sort in the literature is undoubtedly a consequence of the paucity of the data and the difficulties in correlating data obtained from different types of test. The stiffness models described in the paper provide a convenient framework for conducting comparative studies of this sort.

## 2 Methods

An experimental program has been completed at Oxford University, UK, on the use of an improved form of the spinning lens test, originally devised by Fisher (1971), to investigate the stiffness of the human lens. Full details of the design of the test rig, the experimental methods and the data analysis protocols that have been employed to conduct this test program are given in Section 2 of the methods paper. These details are summarised below.

The test rig consists of a vertical rotor driven by a variable-speed DC motor. A detachable lens support ring is mounted at the top of the rotor. The lens to be tested is placed carefully on the lens support, anterior side uppermost. It is manipulated, using ophthalmic spears, to ensure that the polar axis of the lens coincides with the axis of rotation. The lens is then spun, typically at 1000 rpm.

The test is based on the principle that when the lens is spun by the rotor the deformation induced can be related to the (known) centripetal forces to determine the mechanical properties of the lens substance. Data on these deformations are used in conjunction with a computational model of the spinning lens to infer values of lens shear modulus parameters. Tests are conducted on intact lenses and also lenses that have had their capsules removed carefully. Data on lens shear modulus are determined solely from the de-capsulated tests.

The geometry of the lens outline is captured using a digital imaging system that is synchronised with the angular position of the rotor. This system allows images to be collected, rapidly, at 8 equally-spaced angular orientations while the lens is spinning at a desired speed. These images are used to determine an averaged axisymmetric cross-section for the lens (termed the ‘target outline’). Data are also collected when the lens is rotated sufficiently slowly for centripetal forces to be negligible; these data are used to define a ‘reference outline’. This reference outline is used to generate an axisymmetric finite element mesh that is then employed in the optimization process to determine values of shear modulus that provide a best fit between the lens outline computed using the finite element analysis and the target outline obtained from the experiment.

The lens substance is characterised in the finite element analysis by a neo-Hookean constitutive model with the strain energy function, Ψ(C):

(1) $$\Psi \left(C\right)=\frac{\mu }{2}\left(\text{tr}\left[J^{\frac{-2}{3}}C\right]-3\right)+\frac{\kappa }{2}{\left(J-1\right)}^{2}$$

where **C** is the right Cauchy–Green tensor, *J*^2^ = det **C** and *μ* and *κ* are material constants that are allowed to vary spatially within the lens. The lens substance is assumed incompressible and, on this basis, the parameter *κ* is set to be a suitably large number (in comparison with *μ*). In the data analysis procedures used in these tests, the value of *κ* is set to *κ* = 100 *μ*. On this basis, the constitutive behaviour at a material point is determined by a single parameter, *μ*. Since this parameter is closely related to the value of shear modulus as conventionally defined in linear elasticity, the parameter *μ* is referred to in this paper as shear modulus and given the symbol *G*.

**Fig. 1:**
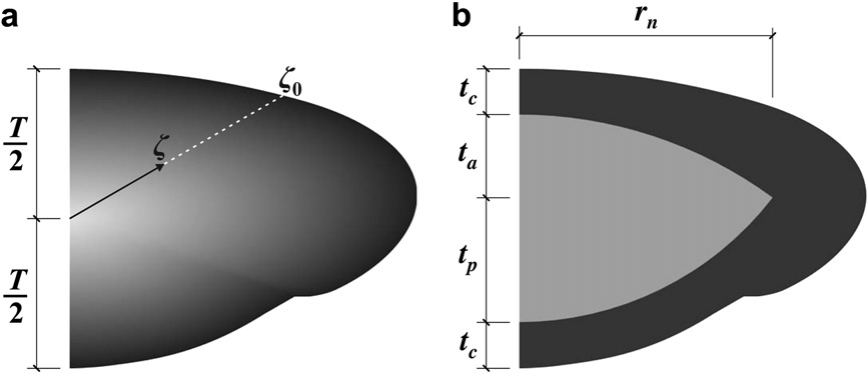
Spatial variation functions (SVF) shown on the cross section of L038A. (a) Model E and (b) Model D. The dimension *T* is the axial thickness of the lens. The dimensions adopted for Model D are: *r*_n_ = 3.45 mm, *t*_a_ = 1.132 mm, *t*_p_ = 1.698 mm. The dimension *t*_c_ is determined by the total axial thickness, *T*, of the particular lens being modelled. Note that the indentation on the posterior lens surface is caused by the support ring.

Inferences on shear modulus values are based on the assumption that the shear modulus varies spatially within the lens. Three separate representations, termed ‘spatial variation function’ (SVF), were adopted to represent the spatial variation of shear modulus in the lens (see Section 2.8 of the methods paper).^1^ One of these models, termed ‘Model H’, takes the simple approach of representing the stiffness of the lens substance by a single value of shear modulus. In another model, termed ‘Model E’, the shear modulus, *G*, of the lens substance is represented by an exponential function of the form:

(2) $$G=\alpha \exp\left(\frac{\beta \zeta }{\zeta_{0}}\right)$$

where *ζ* is the distance of a particular material point from the mid-point of the lens, *ζ*_0_ is the distance to the lens outline along a ray passing through the point, see Fig 1a, and *α*, *β* are parameters describing the model. (Note that this equation is displayed incorrectly in Eq. (1) of the methods paper due to a typographical error). For the purposes of the current paper, Eq. (2) is expressed in the alternative, equivalent, form:

(3) $$G=G_{0}^{\left(1-p_{\text{r}}\right)}G_{1}^{p_{\text{r}}}$$

where *p*_r_ is ‘relative position’ given by:

(4) $$p_{r}=\left(\frac{\zeta }{\zeta_{0}}\right)$$

The parameter *G*_0_, is the shear modulus of the lens at the centre (*p*_r_ = 0) and *G*_1_ is the shear modulus at the outer edge (*p*_r_ = 1). In the third model, termed ‘Model D’, the nucleus and cortex are represented by separate, homogeneous, materials. The shear modulus of the nucleus is *G*_N_ and the shear modulus of the cortex is *G*_C_. The dimensions of the nucleus that are adopted in Model D are given in the caption to Fig. 1.

The parameters in each of the three SVFs defined above are determined, for each individual de-capsulated lens, on the basis of an automated optimization procedure. For each lens, a bespoke axisymmetric finite element mesh is generated from the reference outline (e.g. see Fig 8 of the methods paper). This mesh is used as the basis of a finite element analysis of the test. An optimization procedure is used to determine the values of the parameters defining each SVF that provide a best fit between the target outline and the lens geometry computed using the finite element analysis.

When conducting this finite element inverse analysis, it is necessary to make an assumption about the boundary condition between the lens and the support ring. Two approaches have been used in the current work. In one of these, the lens is assumed to remain fixed to the support ring as a consequence of friction, adhesive and surface energy effects; this is termed ‘fully-fixed’ support (or constraint F). In the other assumption, the lens is assumed to be able to slide over the support; this support condition is termed ‘smooth’ (or constraint S).

## 3 Lens testing program

### 3.1 The tested lenses

A total of 117 lenses from donors aged from 12 to 87 years were received for testing from the Bristol Eye Bank between the 23rd of August 2007 and the 13th of August 2009. Twelve of these lenses were not tested as a consequence of either major damage to the lens during transit or for local operational reasons. Thirty-seven of these lenses were obtained during the early stages of the project and were used to assist in the development of the design of the test rig and the experimental method. Data from this ‘development’ group are not included in the current paper.

Individual lenses in the remaining group of 71 lenses were subjected to spinning tests using the test rig design and experimental protocol described in the methods paper. All lenses (in both intact and de-capsulated form) were subjected to primary tests as specified in Table 1. The standard procedure that was adopted was that data on shear modulus were determined from the images collected in Test B of the primary test sequence. After the primary test sequence had been completed, some of the lenses were subjected to further secondary tests.

Individual lenses are referred to in this paper by a label consisting of ‘L’, followed by a three digit number referencing to the donor and a suffix of ‘A’ or ‘B’ to distinguish the two lenses from the same donor (for example lens L038A). The donor numbers employed in this paper are uncoupled from the identification codes employed by the eye bank. The numerical order of the three digit donor number does, however, correspond to the order in which the lenses were tested. A complete specification of the lenses that were investigated in this test program, together with selected experimental results, is given in the supplementary data.

It should be noted that the data on lens age provided to us by the Bristol Eye Bank were rounded down to the nearest integer. This means that a lens of stated age 45 years could have been taken from a donor aged between 45 and 46 years (less one day). Users of the age-stiffness models described later in the paper (which are based on these rounded down values of age) may wish to take this into account in any application of the model to a lens of a particular age.

### 3.2 Exclusion criteria and Set $\bar{G}$

Although 71 lenses were subjected to the standard spinning test procedure, data from 42 of these were excluded on the basis of the three criteria set out below. The remaining data set of 29 lenses was adopted for detailed analysis of shear modulus parameters. This set of 29 lenses is termed the ‘good quality set’ and is referred to in this paper as Set $\bar{G}$. The lenses in Set $\bar{G}$ are from donors aged between 12 and 58 years with a mean of 40.3 years. For this set of lenses, the average time between death of the donor and spinning lens test procedure was 74 hours with minimum and maximum values of 47 hours and 110 hours respectively. All of the shear modulus data presented in this paper are determined from the 29 lenses in Set $\bar{G}$.

Lenses were excluded from Set $\bar{G}$ on the basis of the following three criteria:

(a) *Mechanical damage*. Lenses were excluded if they suffered mechanical damage during transit, storage or testing. The most frequent cause of damage during the test process was associated with the de-capsulation procedure. A typical occurrence was that a cohesive strip of cortex fibre cells came away with the capsule leaving a depression in the surface of the lens substance. Other forms of damage included the lens accidentally being dropped or being damaged by the lens support ring while it was being manipulated.

All lenses for which mechanical damage was suspected were excluded from Set $\bar{G}$. Any lenses that had been accidentally dropped during testing were excluded even when there was no visible evidence of mechanical damage. Eleven lenses were excluded solely on this basis.

(b) *Surface fluid*. The presence of fluid on the surface of the lens prevents accurate analysis of the test because the fluid obscures the true outline of the lens. Ophthalmic spears were used to absorb fluid from the lens surface when it is positioned on the support ring, but in some cases, detailed analysis of the images collected from the test indicated that some fluid remained on the surface. (When subjected to spinning at 1000 rpm or faster, for example, fluid on the lens surface generally forms a characteristic bulge at the lens equator). Note that since the lens stiffness data are determined from the de-capsulated tests, tests in which the intact lens suffered from surface fluid, but the de-capsulated test did not, were not excluded.

Surface fluid was found to have influenced 10 lenses which were otherwise of an acceptable quality. These 10 lenses were excluded from Set $\bar{G}$.

(c) *Lens swelling*. Lenses are prone to *post-mortem* swelling following death. It is plausible (although to the authors' knowledge this has not been tested experimentally) that swelling of the lens may influence its measured stiffness. In view of this, excessively swollen lenses were excluded from Set $\bar{G}$.

A decision on whether a lens is excessively swollen is based on observed values of lens aspect ratio, *R*_A_, which is defined:

(5) $$R_{\text{A}}=\frac{D}{T}$$

where *T* is the axial thickness and *D* is the equatorial diameter; both dimensions were determined from images of the intact lens when placed on the support ring. As the lens swells, the aspect ratio tends to decrease (Augusteyn, 2008). According to Augusteyn an isolated intact adult lens typically has an aspect ratio of between 2.2 and 2.3. An aspect ratio of less than 2.0 would suggest that swelling has occurred. Augusteyn also notes that unswollen young lenses tend to exhibit lower values of aspect ratio than older unswollen lenses.

In the current study, the aspect ratio for each lens is determined from the reference outline computed for the first reference test (*A*_ref_ in Table 1) on the intact lens. In three cases, surface fluid rendered the first reference test unusable. In these cases, since surface fluid prevents the accurate determination of the lens outline, the aspect ratio values for use in Eq. (5) were computed from another reference test on the intact lens.

Lenses were excluded on the basis of swelling if the donor was of age 25 years or more and *R*_A_ < 1.95. No attempt was made to exclude lenses of age less than 25 years on the basis of a swelling criterion. This is on the basis that young lenses, being less stiff, tend to experience a significant decrease in aspect ratio as a consequence of the deformations induced by the lens support ring. In these cases, observed measurements of aspect ratio may be an unreliable indicator of the state of the lens. Also, since the aspect ratio of young lenses tends to be less than those from mature adults (Augusteyn, 2008) a low aspect ratio in young lenses may not necessarily be indicative of swelling.

**Fig. 2:**
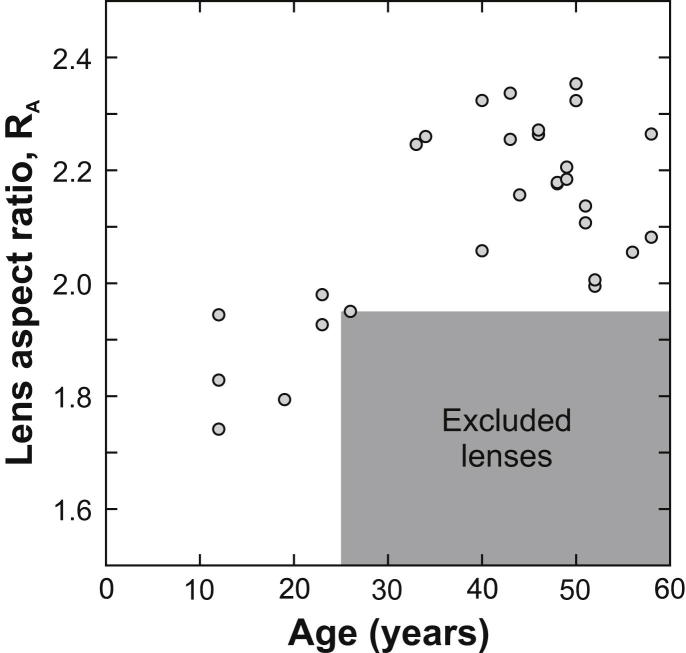
Values of lens aspect ratio for lenses in Set $\bar{G}$. Lenses with age and aspect ratio falling in the grey region were excluded from Set $\bar{G}$ on the assumption that they suffer from excessive *post-mortem* swelling.

A total of 21 lenses were excluded from Set $\bar{G}$ on the basis of the swelling criterion described above. The aspect ratios of the lenses that were ultimately included in Set $\bar{G}$ are plotted in Fig. 2.

## 4 Results

The test program provided an extensive set of data on intact and de-capsulated lenses. Analysis of these data was concerned principally with the use of the de-capsulated lens images to infer values of the lens shear modulus parameters.

Shear modulus data obtained from Set $\bar{G}$ were used to develop models for the development of lens shear modulus with age. Models of this sort are referred to in this paper as ‘age-stiffness models’. In addition, detailed analysis has been conducted on three representative lenses; these lenses are a 33-year lens L038A, a 43-year lens L039B, and a 50-year lens L056B. (Note that L038A is the same 33-year lens that is described in the methods paper).

### 4.1 Load-deformation responses

**Fig. 3:**
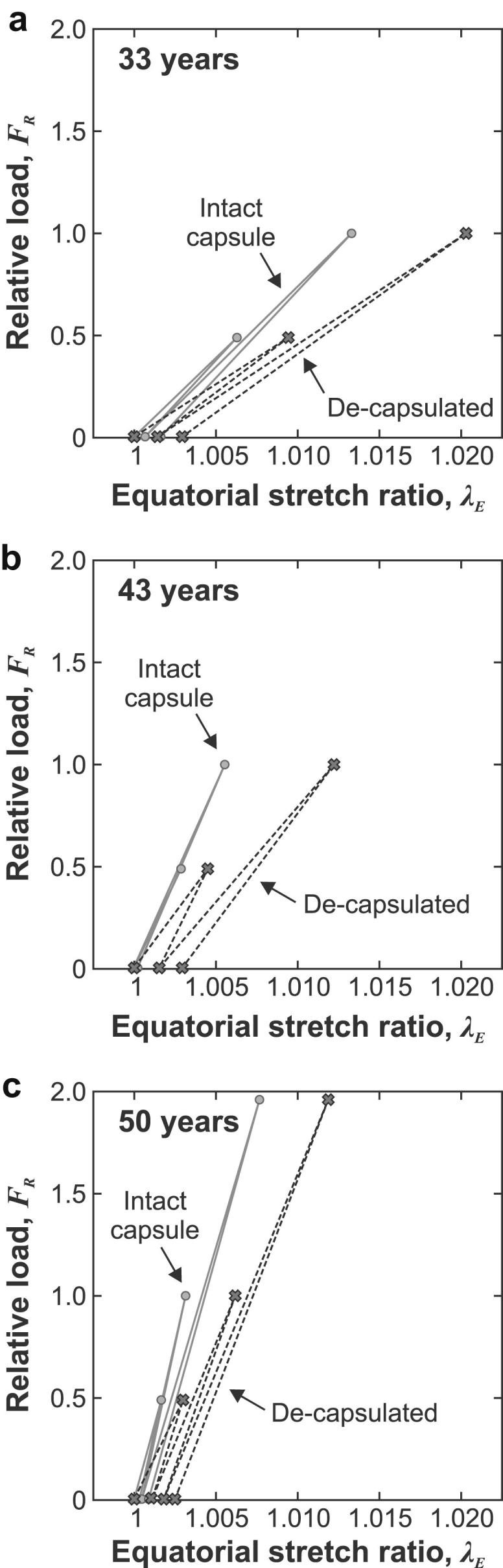
Relative load – equatorial stretch data for the three representative lenses. Note that the 50-year lens was subjected to an extended test sequence which involved spinning the lens to 1400 rpm at the end of the primary sequence.

The body forces induced in the lens as a consequence of its rotational motion are quantified using the parameter ‘relative load’, *F*_R_, which is defined as:

(6) $$F_{\text{R}}=\frac{\omega^{2}}{\omega_{\text{o}}^{2}}$$

where, *ω*, is the current rotational speed and the reference rotational speed, *ω*_o_, corresponds to 1000 rpm. The deformations induced in the lens are conveniently quantified by the equatorial stretch ratio, *λ*_E_, which is defined by:

(7) $$\lambda_{\text{E}}=\frac{D}{D_{A_{\text{ref}}}}$$

where *D* is the current diameter (determined from the target outline) and $D_{A_{\text{ref}}}$ is the diameter determined from Test *A*_ref_. Data on the relative load – equatorial stretch ratio response for the three representative lenses are shown in Fig. 3.

The slope of the relative load-equatorial stretch responses for each lens provide a general indication of the overall stiffness of the lens. It is clear from Fig. 3 that this slope is approximately the same for each lens at each test speed. This suggests that the lens behaves in an approximately linear manner over the range of rotational speeds adopted. It is also clear that, in all cases, the overall stiffness of the de-capsulated is less than that observed for the intact lens. The presence of the capsule (as would be expected) influences the observed response of the lens in the test. The experimental technique employed in the current tests (in which the stiffness of the lens substance is determined on the basis of measurements on the de-capsulated lens) removes the confounding influence of the lens capsule.

These lenses all display unrecovered deformation following each loading and unloading cycle. The origin of these residual deformations is uncertain. They may be associated with time-dependent behaviour of the lens substance (e.g. as observed in the cyclic tests described by Weeber et al., 2007; Weeber et al., 2005) or unrecovered slippage of the lens at the support. The current analysis of the test examines the response on the basis of an elastic model (in which time-dependent effects are explicitly excluded). To ensure a consistent basis for conducting the inverse analyses, the reference configuration used in the finite element inverse analysis of Test B (which is the standard procedure used to infer the shear modulus) is based on a combined set of observed lens outlines from the reference tests *B*_ref_ and *C*_ref_, respectively before and after Test B (see Table 1 and also the methods paper, Section 3).

### 4.2 Bulk deformations of intact and de-capsulated tests

**Fig. 4:**
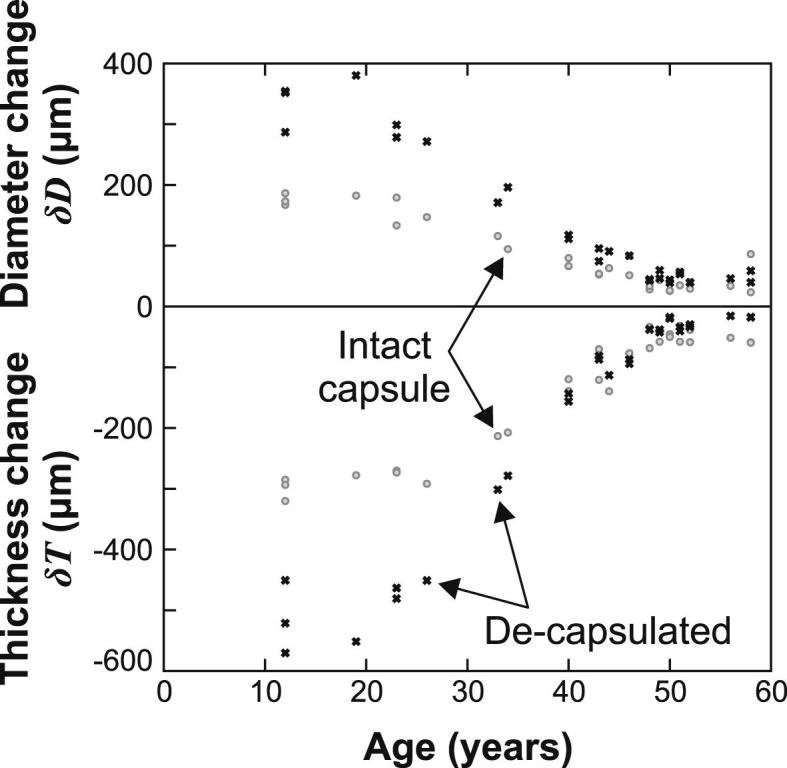
Change in lens diameter and polar thickness of the lenses of Set $\bar{G}$ when spun at 1000 rpm.

Data on changes in equatorial diameter, *δD*, and polar thickness, *δT*, for all of the lenses in Set $\bar{G}$, are shown in Fig. 4. These data are determined from the target outline for Test B with respect to the relevant reference configuration. The intact lens results may be compared with the data in Fig. 7 of Fisher (1971). It is seen that the current intact lens results show similar trends to the Fisher data (e.g. tendency for displacements to decline with increasing age). However, data plotted in Fisher (1971) exhibit rather more scatter than our own results – this is perhaps a consequence of the improved systems for image capture and analysis employed in the present tests.

For young lenses (i.e. less than about 35 years old) the data in Fig. 4 indicate that the capsule has a consistent and appreciable effect on restricting the deformations induced in the test. With increasing age, the capsule is seen to have a diminishing influence. For three of the older lenses the deformations in the intact lens were actually greater than those in the de-capsulated lens. This pattern is consistent with a view of the lens in which the lens substance stiffness increases very substantially during life but that the stiffness of the capsule changes only moderately, if at all (as is evident, for example, in the data of Krag and Andreassen, 2003). The standard rotational speed of 1000 rpm adopted in these tests induces changes of lens diameter change of about 300 microns in the youngest de-capsulated lenses (age 12–20 years) reducing to a value of about 50 microns for lenses aged 50 years and older. These lens diameter changes are rather less (but of comparable magnitude) than those expected during the natural accommodation process (e.g. on the basis of the MRI data of Strenk et al., 1999; Sheppard et al., 2011). This supports the assumption, implicit in the experiment, that the induced deformations at 1000 rpm are not large enough to cause mechanical damage to the lens.

### 4.3 Assumed lens support condition

Shear modulus data for the lens substance have been calculated for all of the de-capsulated lenses of Set $\bar{G}$ using the analysis procedures described in the methods paper. The principal results are those obtained from Test B in the primary test sequence conducted at 1000 rpm. For each lens, analyses were conducted in which the interface between the lens and the support is assumed to be fully-fixed (constraint F) or smooth (constraint S) (see Section 2.8 of the methods paper). Analyses were also conducted for each of the three forms of SVF employed in the data processing protocol. This provides six different descriptions of the shear modulus of the lens substance for each lens.

Values of shear modulus data determined using Model H increase very substantially with age. The data, however, are relatively insensitive to the nature of the support condition assumed in the inverse analysis. Shear modulus values calculated using constraint S are, on average, about 12% greater than the values calculated using constraint F. This difference is small compared with the very substantial stiffness changes that occur with small changes in age. In contrast, the results of the inverse analysis with the two non-homogeneous models (Model D and Model E) exhibit a significant influence of the support condition on the inferred values of shear modulus.

**Fig. 5:**
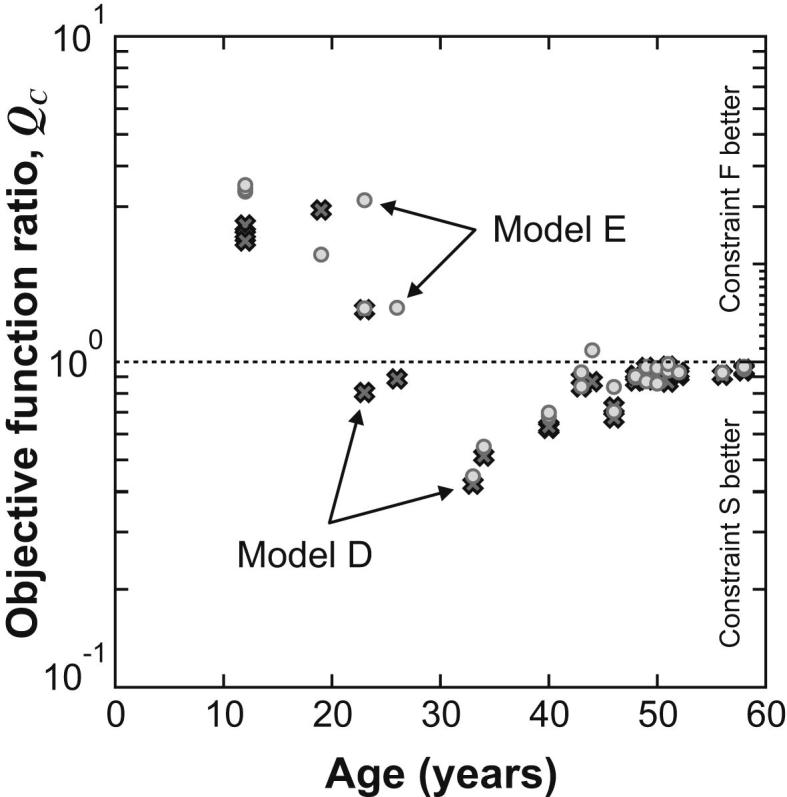
The objective function ratio, *Q*_C_, plotted on a logarithmic scale. Model E data are shown as circles and Model D data are shown as crosses. If *Q*_C_ > 1 then constraint F provides the best match with the reference outline. Conversely, if *Q*_C_ < 1 then constraint S provides the best match.

The photographs of the spinning test provide little direct information that could be used to determine which constraint assumption is the most appropriate for modelling the contact with the lens support ring. However, the optimization procedure employed to determine the shear modulus parameters does provide a metric on how well the target lens outlines are reproduced by each constraint, for each lens, in the form of the minimum value of the parameter *A*_*i*_ (which is the area enclosed between the computed and target outlines, see Section 2.8 of the methods paper). To investigate the relative merits of the F and S constraints for each particular lens, the objective function ratio *Q*_C_ is defined where:

(8) $$Q_{\text{C}}=\frac{{\left(A_{i}\right)}_{\text{S}}^{\min}}{{\left(A_{i}\right)}_{\text{F}}^{\min}}$$

and ${\left(A_{i}\right)}_{\text{S}}^{\min}$, ${\left(A_{i}\right)}_{\text{F}}^{\min}$ are the minimum values of the parameter *A*_*i*_ for the S and F constraint conditions respectively. Data on *Q*_C_ for all of the lenses in Set $\bar{G}$ are plotted in Fig. 5.

The values of *Q*_C_ plotted in Fig. 5 show a broadly consistent pattern: constraint F provides a better match to the experiment for 12 of the 14 analyses when applied to lenses younger than 30 years, while constraint S provides a better match for 43 of the 44 analyses when applied to lenses of 30 years or older. It seems plausible that the younger lenses are more constrained by the support ring as a consequence of their tendency to sit lower and to deform significantly around the support under the action of gravity loading; this is consistent with the observation that constraint F appears to provide a better fit with the data than constraint S for the younger lenses.

The most appropriate condition at the support for any particular lens probably lies between the extremes of the conditions implied by constraints F and S. However, in the absence of further detailed information, to infer values of shear modulus data, lenses of age less than 30 years were analysed using constraint F and lenses of age 30 years and older were analysed using constraint S. This standard procedure to model the support boundary condition has been adopted in the calculation of all of the shear modulus data described in this paper.

### 4.4 Shear modulus data for the representative lenses

**Fig. 6:**
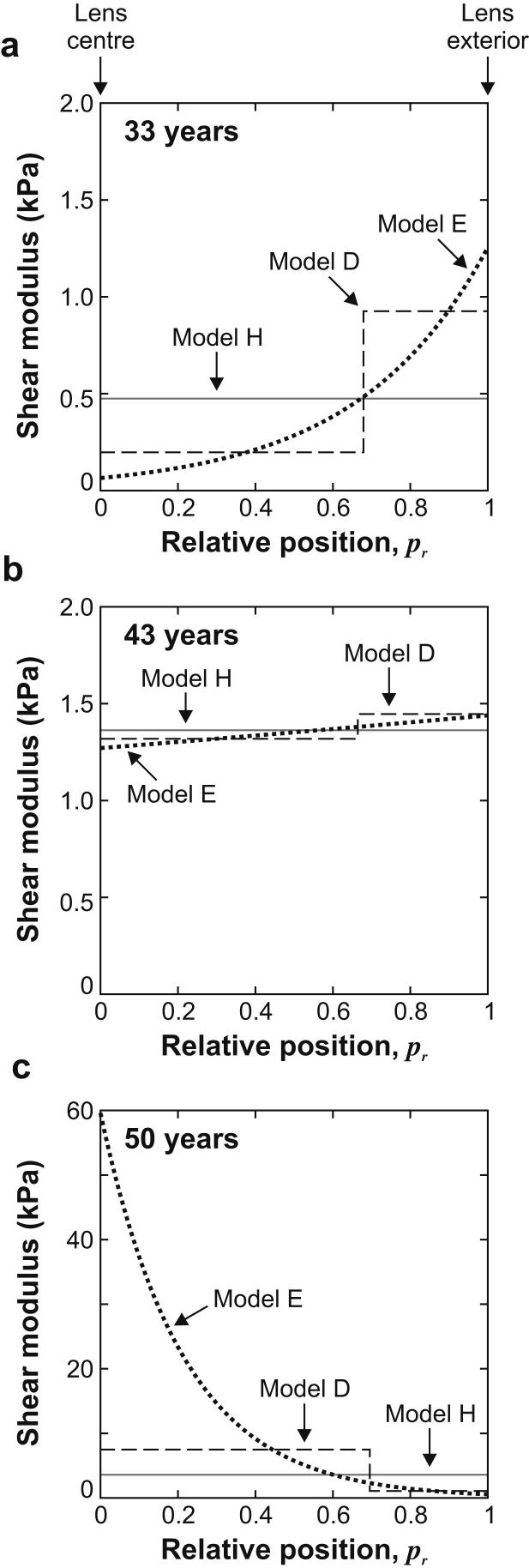
Shear modulus profiles for the three representative lenses. Numerical values of the data are given in Table 2.

The optimum computed stiffness profiles for each of the three forms of SVF for the three representative lenses are shown in Fig. 6; the relevant shear modulus parameters are given in Table 2. In these plots the step stiffness change in Model D is plotted at the mean relative position of the transition from the nucleus to the cortex. For the 33-year lens the two non-homogeneous models indicate that the nucleus is less stiff than the cortex. This situation is reversed for the 50-year lens, for which the nucleus is stiffer than the cortex. The 43-year lens provides an interesting example of the intermediate case where the lens is approximately homogeneous (i.e. the nucleus and cortex have similar values of shear modulus). In all cases, as expected, the Model H data are intermediate between the extreme values of both Models D and E.

It is noted from Fig 6c that for the 50-year lens the inferred shear modulus at the centre of the lens is substantially larger for Model E than for Model D. This tendency of the Model E data to provide relatively large values of central stiffness for older lenses is a consequence of the exponential form of the SVF. When the centre of the lens is substantially stiffer than the outer and intermediate regions, the computed response of the spinning lens outline becomes insensitive to the precise numerical value of stiffness at the centre. When the optimization process is applied to such a lens it will tend to provide appropriate stiffness values for the outer and intermediate regions of the lens, while the central value will be determined principally by the mathematical form of the SVF. In these cases, the precise value of stiffness computed at the centre of the lens is poorly conditioned. When Model E is applied to the older lenses (in which the nucleus is substantially stiffer than the cortex) the exponential nature of the functionproduces central stiffness values that are thought to be unrealistically large. For lenses than are older than about 45 years, therefore, it is thought that the central region of the lens is likely to be better represented by Model D than Model E. This issue does not arise for the younger lenses for which the centre of the lens is less stiff than the cortex.

**Fig. 7:**
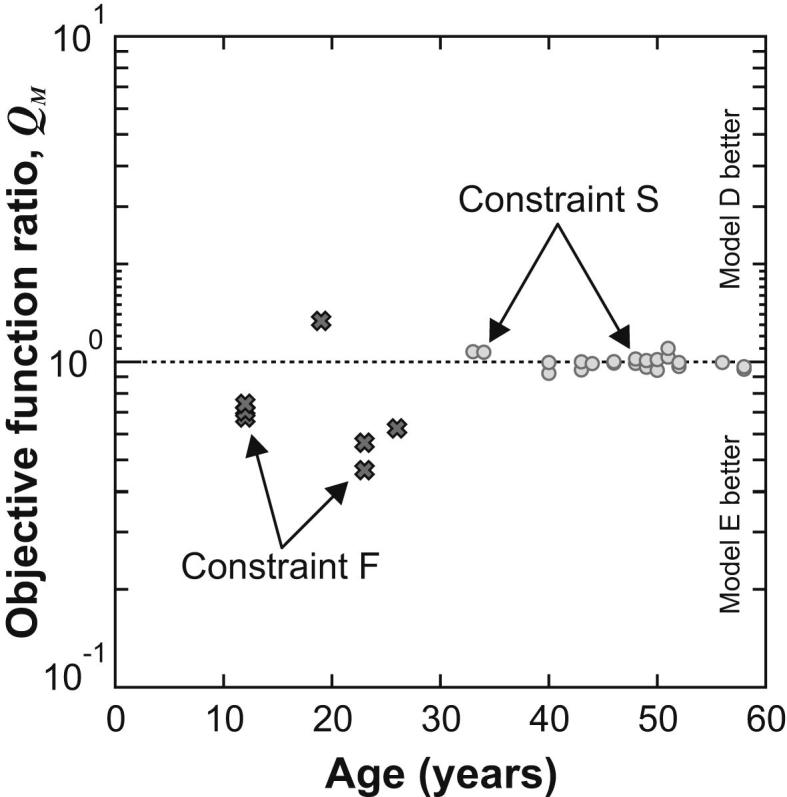
The ratio, *Q*_M_, of the optimum of the objective function obtained using Model E to that obtained using Model D, plotted on a logarithmic scale. Values are obtained using the fixed support (constraint F, data shown as crosses) for ages less than 30 years and the sliding support (constraint S, data shown as circles) for lenses aged more than 30 years. If *Q*_M_ > 1 then Model D provides the best match with the reference outline. Conversely, if *Q*_M_ < 1 then Model E provides the best match.

The relative performance of Models E and D has been examined in terms of quantity *Q*_M_ which is defined:

(9) $$Q_{\text{M}}=\frac{{\left(A_{i}\right)}_{\text{E}}^{\min}}{{\left(A_{i}\right)}_{\text{D}}^{\min}}$$

where ${\left(A_{i}\right)}_{\text{E}}^{\min}$, ${\left(A_{i}\right)}_{\text{D}}^{\min}$ are the minimum values of the parameter *A*_*i*_ for models E and D respectively. A value of Q_M_ that is less than one indicates that Model E provides a better fit with the experiment, while a value greater than one indicates that Model D provides a better fit with the experiment. Fig. 7 indicates that Model E provides a better fit for the young lenses (i.e. less than 30 years) and models D and E are equally good for lenses that are older than 30 years. The generally better performance of Model E for the younger lenses may indicate that the form of the nucleus assumed in Model D is inappropriate for these lenses, either because a mechanically distinct nucleus does not exist, or because its assumed size and/or shape is incorrect.

### 4.5 Age-stiffness models for the lens

The relations between lens age and the stiffness parameters of the three forms of SVF (models H, D, and E ) for all of the lenses in Set $\bar{G}$, tested at 1000 rpm, can usefully be represented by analytical best-fit functions. These functions are referred to in this paper as ‘age-stiffness models’.

**Fig. 8:**
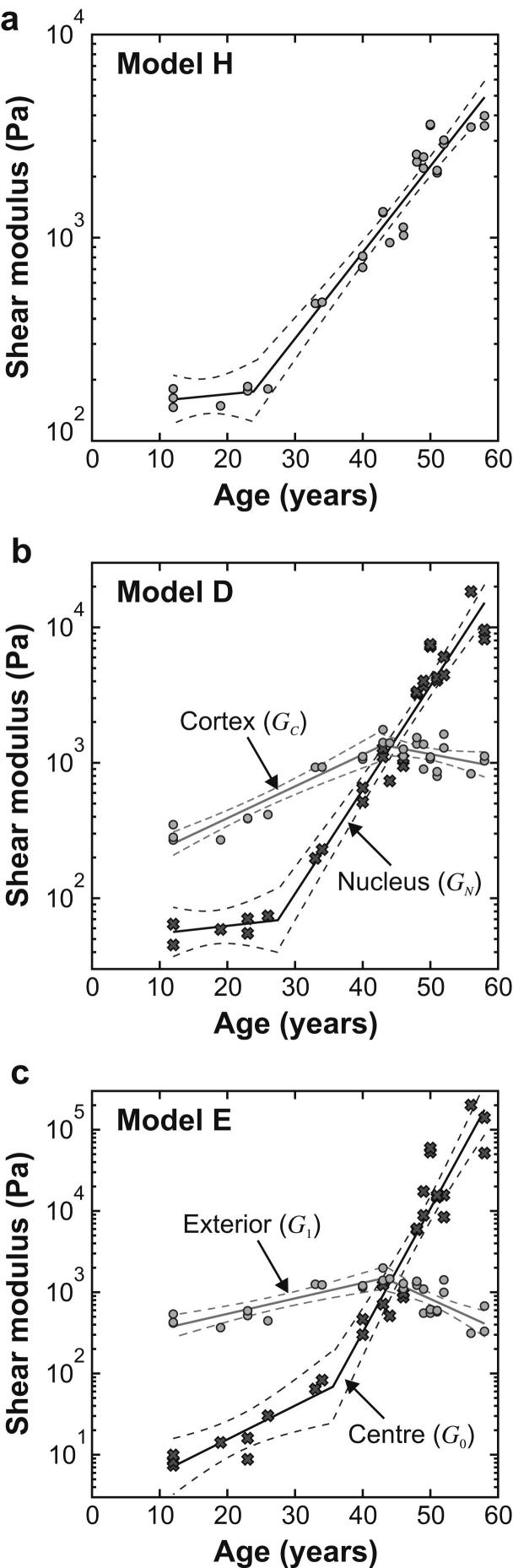
Age-stiffness relationships for (a) Model H (b) Model D and (c) Model E. The dashed lines indicate the 95% confidence bounds for the fitted function. Parameters are specified in Table 3. Note that shear modulus is plotted in a logarithmic scale.

Age-stiffness models have been determined on the basis of the following functional form:

(10) $$\log_{10}\left(\frac{f}{c}\right)=\left\{ \begin{array}{l} b_{1}\left(A-A^{*}\right)\;A\leq A^{*}\\ b_{2}\left(A-A^{*}\right)\;A>A^{*} \end{array}\right.$$

where *f* is the particular shear modulus parameter being represented and *A* is age. This form of model provides a piece-wise linear relationship between age and logarithm of the shear modulus. The model parameters *A*^*∗*^, *b*_1_, *b*_2_ and *c* are determined to provide a best fit with the data inferred from the experiment. The MATLAB utility *cftool* was used to calculate the optimum values of the model parameters and the corresponding 95% confidence intervals for the fitted function, both calculated from the logarithms of the stiffness values. Model parameters are specified in Table 3. The models are plotted, together with the data, in Fig. 8.

The stiffness data all display a substantial rate of increase with age. The ‘doubling time’, *T*_2_, is defined as the age change needed for a particular stiffness coefficient to double in value. For the linear portions of the model, the doubling time is given by:

(11) $$T_{2}=\left\{ \begin{array}{l} \left(\log_{10}2\right)/b_{1}\;A\leq A^{*}\\ \left(\log_{10}2\right)/b_{2}\;A>A^{*} \end{array}\right.$$

Data in Table 3 show, for example, that the doubling time for the nucleus in Model D, for ages greater than 27.5 years is about 4 years. Data presented in this way provide a stark indication of the very substantial stiffness changes that occur in the nucleus with increasing age.

## 5 Reliability of the data

### 5.1 Shear modulus data determined at different rotational speeds

**Fig. 9:**
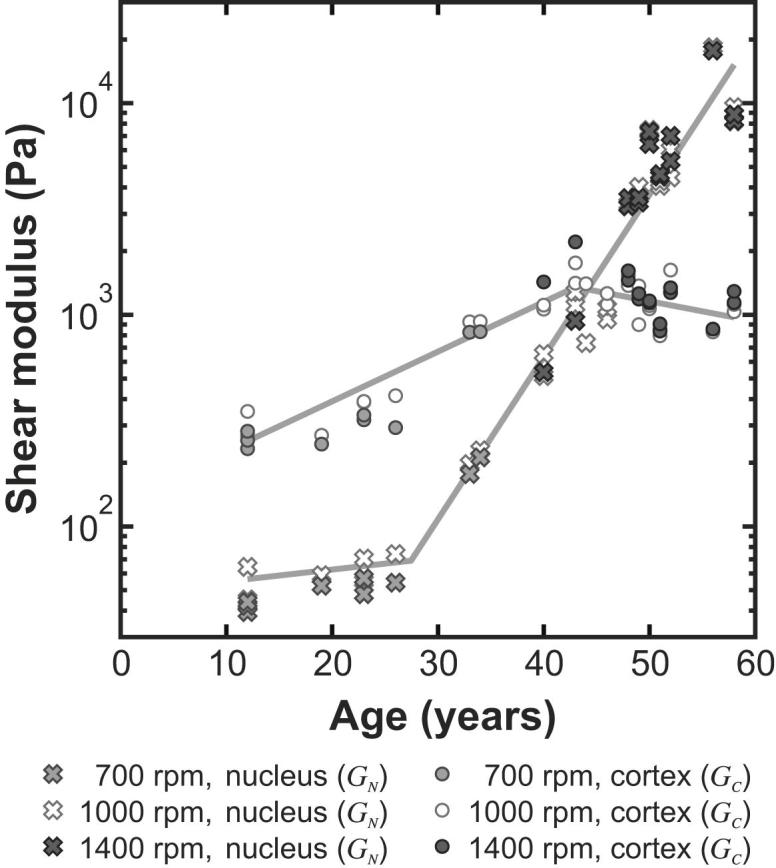
Shear modulus data inferred for Model D at 700 rpm, 1000 rpm and 1400 rpm. The solid lines show the age-stiffness model for Model D defined in Eq. (10) and Table 3.

The shear modulus data presented in Fig. 8 were all determined from experiments conducted in Test B (i.e. at 1000 rpm) of the primary test sequence (see Table 1) on the de-capsulated lens. Inferences on lens stiffness have also been computed for the images collected in Test A (when the lens is spinning at 700 rpm). In some cases, particularly for the older lenses, secondary tests in which the lens was spun at a rotational speed of 1400 rpm were conducted after the primary test sequence. Shear modulus data for Model D determined from tests conducted at these different rotational speeds are plotted in Fig. 9.

The stiffness parameters obtained from tests at 700 rpm and 1400 rpm broadly agree with those obtained at 1000 rpm. The shear modulus parameters obtained from the 700 rpm tests, however, are all lower than those from the corresponding 1000 rpm tests, with the value for *G*_N_ determined at 700 rpm being, on average, 0.81 of the 1000 rpm result, and the value for *G*_C_ determined at 700 rpm being, on average, 0.85 of the corresponding 1000 rpm result. However, the behaviour at 1400 rpm does not differ from that at 1000 rpm in the same systematic manner. The average value of *G*_N_ at 1400 rpm coincides with that at 1000 rpm and the value for *G*_C_ at 1400 rpm is, on average, 1.07 times the value determined at 1000 rpm.

The variation in the calculated values of shear modulus determined at different test speeds is relatively small compared to the change in the shear modulus parameters associated with modest difference in age. The response of the substance of the older lenses appears to be essentially linear up to 1400 rpm, while the substance of the younger lenses may indicate a slightly non-linear response (with the stiffness increasing with strain). It is also plausible, however, that the differences between the results of the 700 rpm and 1000 rpm tests observed for the younger lenses, could be explained in terms of a preconditioning effect.

### 5.2 Precision of the optimization procedure

The optimization procedure provides a precise value for the shear modulus parameters that provide a best fit between the finite element model and the observed geometry changes that develop in a given lens during the test. However, it should be noted that the procedures employed to determine the shear modulus parameters are necessarily subject to various experimental and modelling errors. These errors influence the accuracy of the computed stiffness parameters in ways that can be quantified, as outlined below.

**Fig. 10:**
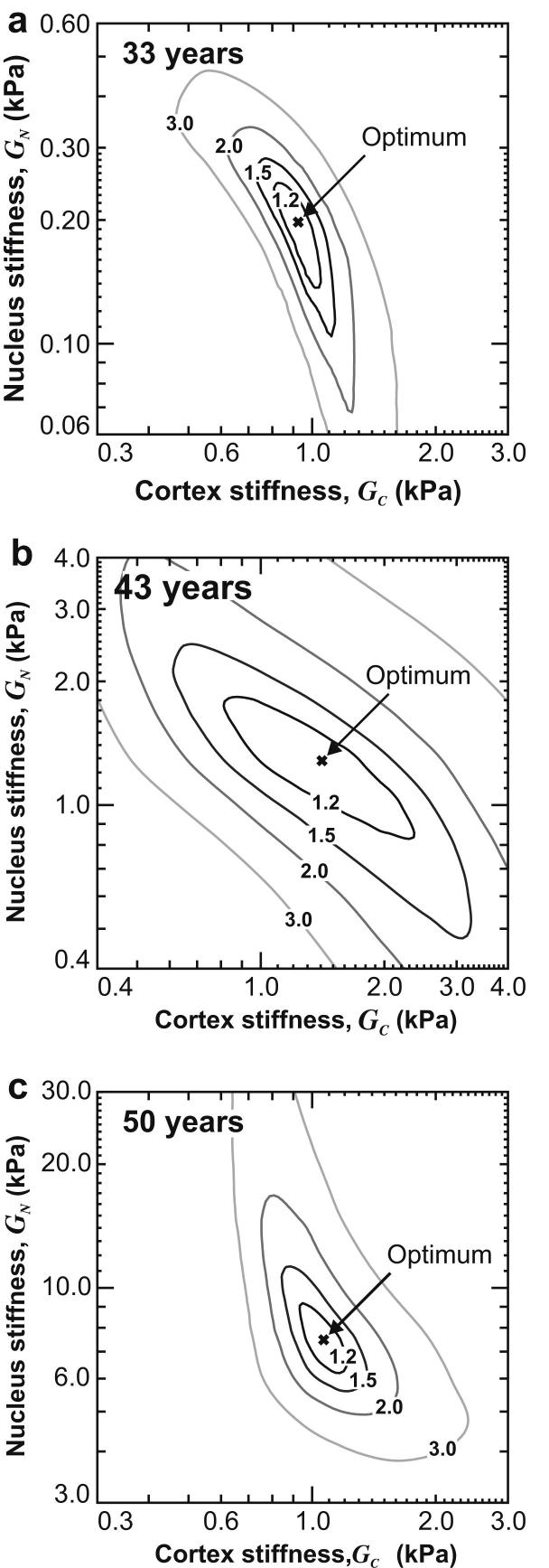
Contours of *Q*_A_ for the three representative lenses for Model D.

For a given set of parameters, the error in fitting the computed outline to the target outline is represented by the area difference parameter *A*_*i*_. A quality of fit parameter *Q*_A_ is defined:

(12) $$Q_{\text{A}}=\frac{A_{i}}{{\left(A_{i}\right)}_{\min}}$$

where (*A*_*i*_)_min_ is the area difference parameter when the optimum values of stiffness parameters are adopted in the finite element analysis of the test. Contour plots of *Q*_A_ are shown in Fig. 10 for the three representative lenses, for Model D. These plots provide a rough indication of the likely influence of modelling and experimental errors on the computed values of the stiffness parameters. Note that the data plotted in these graphs are defined differently to the error parameter that is plotted in Fig. 12 of the methods paper. The parameter *Q*_A_ is adopted in the current plots on the basis that it provides a direct indication of the likely impact of modelling and experimental errors on the inferred values of stiffness.

The point on each contour plot where *Q*_A_ = 1 indicates the optimum value of the stiffness parameters. The influence of errors inherent in the experimental and numerical procedures used to infer the lens stiffness data can be quantified by inspecting the range of shear modulus values that fall within a particular contour of *Q*_A_. For example, consider the 1.2 contour (which would correspond to an error of 20% in the determination of *A*_*i*_). For the 33-year and 50-year lenses, the 1.2 contour prescribes a relatively small range of plausible values of cortex and nucleus shear modulus. In these cases, since the lens exhibits a very marked non-homogeneity, the inverse analyses procedure adopted in the experimental protocol is able to compute data on stiffness within relatively close bounds.

The wider range of plausible values seen for the 43-year lens in Fig 10(b) arises as a consequence of the fact that for this particular lens the quality of the optimization process (quantified by the error parameter *γ*_E_ defined in the methods paper) is inferior to the quality achieved for the other two lenses. (The error parameter is 0.19 for the 43-year lens compared to 0.07 for the 33-year lens and 0.11 for the 50-year lens).

### 5.3 Drying of the lens

The lens is confined in a humid environment during the test. We considered whether significant moisture loss from the lens occurs during the test and, if so, whether this might affect the inferred values of stiffness. A detailed study of this issue is given in the methods paper, in the context of L038A.

To extend this previous study, secondary tests were conducted on L039B (43-year) and L056B (50-year). These secondary tests employed the same methods that were adopted for the 33-year lens, L038A, as described in the methods paper. To investigate the effects of moisture loss, shear modulus values were determined from the primary 1000 rpm test (typically after spinning the lens for about 8 minutes) and then from a secondary test (typically after spinning the lens for about 19 min at 1000 rpm). The resulting shear modulus data, for Model D, are given in Table 4.

It can be anticipated that drying will tend to increase the stiffness of the exterior of the lens. This is indeed reflected in the stiffness parameters obtained for the representative lenses, as shown in Table 4, where the cortex is on average about 1.09 times stiffer in the secondary test than in the primary test. The difference cannot be unambiguously attributed to the drying of the lens, however, since unrecovered deformations or preconditioning effects may also play a role in the changed response of the lenses observed in the secondary tests. It does, however, suggest that drying has at most a modest effect on the stiffness parameters obtained from the primary tests compared, for example, with the substantial variation seen between individual lenses of a similar age.

## 6 Comparisons with published data

An ambition of the current work is that the shear modulus results will contribute to a body of data that will allow a clearer understanding to be developed, in quantitative terms, of how the stiffness of the lens substance varies with age and spatial position within the lens. If an understanding of this sort is to be achieved, it will be necessary to demonstrate that stiffness measurements on the lens using different types of mechanical test are able to combine to provide an unambiguous pattern. As an initial step, an attempt has been made to compare the current data with those from published measurements available in the current literature.

It is important to note that numerical values of stiffness are only meaningful in the context of the particular constitutive model that is being employed to represent the material behaviour. Published data on lens stiffness are generally based on an isotropic elastic constitutive model (although this is extended to viscoelasticity by Weeber et al., 2007, 2005). Our own shear modulus data, computed on the basis of a nearly incompressible neo-Hookean model, can be compared directly with these previous studies on the assumption that the magnitude of the strains developed in the lens during the spinning test are insufficient to mobilize significant non-linearities in the neo-Hookean model. It should be noted, however, that any shortcomings in the constitutive model employed to represent the mechanical behaviour of the lens substance will tend to cause differences in the values of stiffness parameters determined from different types of test. For example, the lens substance consists of a structured arrangement of lens fibres. It might be expected, therefore, that the mechanical behaviour of the lens will exhibit some form of anisotropy. In this case, any stiffness parameters determined on the basis of an isotropic model will depend to a certain extent on the mode and direction of loading employed in the test.

The exercise described below is presented on the basis of a straightforward comparison of data. In cases where there is lack of agreement between data sets we have not attempted a detailed assessment of the likely reasons for the disparity. The comparisons are presented here simply on the basis that they provide a picture on how our data fit within the general pattern of the current set of published data.

### 6.1 Comparison with Fisher (1971)

In the original spinning lens test of Fisher (1971) the lens is assumed to consist of a distinct nucleus and cortex (as in the current Model D). However, the nucleus is modelled as a sphere to simplify the calculations. In addition, since the tests were conducted on lenses with the capsule intact, the lens substance stiffness data will have been systematically influenced by the presence of the capsule.

**Fig. 11:**
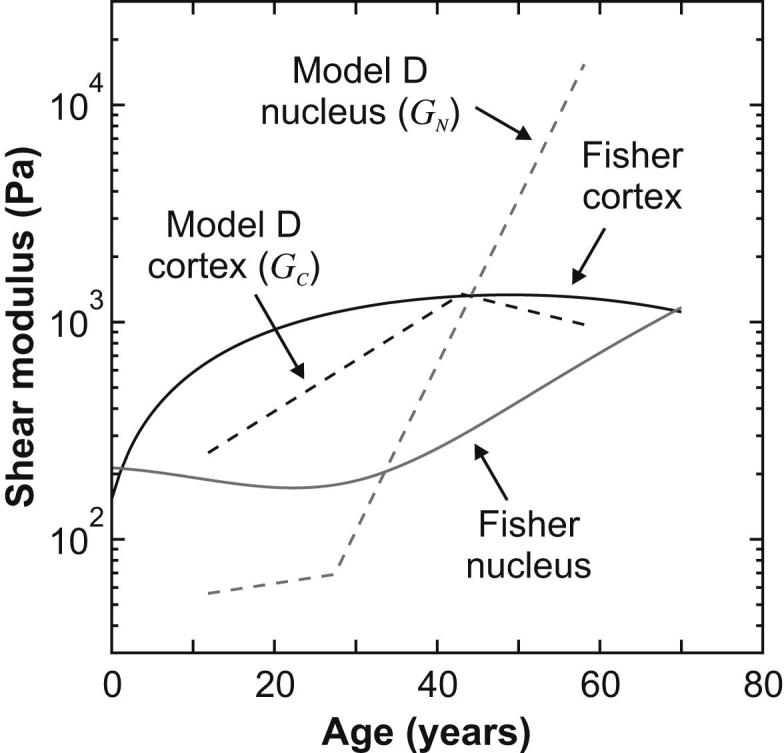
Age-stiffness model for the nucleus and cortex reported by Fisher (1971) (solid lines) compared to the Model D age-stiffness model (dashed lines). The Fisher (1971) models have been translated from Young’s modulus to shear modulus assuming incompressibility and plotted in log_10_-space.

A comparison is shown in Fig. 11 between the age-stiffness model proposed by Fisher (1971) and the current age-stiffness model, for Model D.

In broad terms, the general pattern exhibited by the Fisher (1971) and the Model D age-stiffness models are similar. For young lenses the cortex is stiffer than the nucleus and, with increasing age, both models indicate that the nucleus stiffens more rapidly than the cortex. However, the general magnitude of the changes in stiffness with age, especially in the nucleus, are much greater in the age-stiffness model for Model D than in the Fisher (1971) results. The relatively low changes in stiffness with age implied by the Fisher data is consistent with the likelihood that the presence of the capsule in the Fisher tests had a significant stiffening effect on the younger lenses (e.g. compare the intact and de-capsulated data in Fig. 4.)

### 6.2 Comparison with data in Heys et al. (2004, 2007)

Heys et al. (2004) investigated the mechanical behaviour of previously-frozen human lenses by means of micro-indentation tests conducted on lenses that had been sectioned equatorially. Heys et al. (2007) give data from a similar set of tests, although in this case the lenses were not frozen prior to testing. The data from both sets of tests are presented in terms of a shear modulus. It seems reasonable to consider whether useful comparisons can be made between these micro-indentation data and the shear modulus data presented in the current paper.

According to Heys et al. (2004), the indenter was cylindrical and flat faced, with a diameter of 0.4 mm. Heys et al. (2004) report that ‘*Typically the displacement was of the order of 750 μm*’. Shear modulus values were computed on the basis of the measured load applied to the indenter and the maximal depth of penetration using a standard equation for a rigid indenter on an incompressible elastic half-space.

The micro-indentation data in Heys et al. (2004, 2007) were apparently obtained for the explicit purpose of correlating the physical properties of the lens with other data on water content and biochemistry. The data provide a striking indication of the way in which the mechanical properties of the lens change spatially and with age. However the relatively large penetration depths adopted in the experiment would be expected to generate local strains with magnitudes that exceed (by a very considerable amount) the strains that are relevant to accommodation. For example, when a displacement *δ* is imposed on an incompressible elastic half-space by a rigid indenter of diameter *d*, then from standard solutions from linear elasticity (e.g. Hills et al., 1993) the horizontal (tensile) and vertical (compressive) strain at a typical point located at distance *d*/2 below the centre of the indenter is computed to be 0.3*δ*/*d* and 0.6*δ*/*d* respectively. From the values given in Heys et al. (2004) (i.e. *δ* = 0.75 mm and *d* = 0.4 mm) this gives values of tensile strain of 60% and compressive strain of 120%. These values of strain are more than an order of magnitude larger than typical values of strain developed in the spinning lens test. This suggests that, if any large strain material non-linearity exists, then the data from Heys et al. (2004, 2007) would not be expected to correlate well with the current results. More importantly, however, it seems unlikely that the lens substance is able to sustain tensile strains as high at 60% while remaining intact. Instead, it seems likely that a zone of tensile tearing will develop ahead of the indenter and that, as a consequence, the probe will penetrate the lens substance rather than causing a reversible elastic indentation. Any departure of this sort from elastic behaviour has the consequence that the indentation data cannot, strictly, be interpreted solely in terms of an elastic shear modulus.

It therefore seems inappropriate to attempt a detailed numerical comparison between the indentation data from the Heys et al. (2004, 2007) tests and the current data. It is of interest, however, to compare the general trends in these indentation test data with the results presented in the current paper. The authors’ hold the opinion that the Heys et al. (2004, 2007) indentation data do not represent shear modulus in the strict sense employed in the current paper. To avoid confusion, therefore, in the discussion below, numerical values of the Heys et al. (2004, 2007) indentation data are referred to ‘stiffness index’ rather than using the term ‘shear modulus’.

Qualitatively, the numerical values of stiffness index given in Fig. 5 of Heys et al. (2004) correspond closely to the results from the current study. Plotted values of stiffness index show that for ages less than about 35 years the nucleus is less stiff than the cortex and that for ages greater than 35 years the position is reversed. This corresponds closely to the pattern in Fig. 8(b) and (c). It is noted, however, that in our results the ‘cross-over’ age (i.e. the age at which the nucleus and cortex have the same value of stiffness) is 44 years.

Further comparison between the two sets of data is facilitated by the parameter ‘stiffening ratio’, SR, which is defined:

(13) $$\text{SR}=\frac{\text{Stiffness}\;\text{at}\;\text{age}\;50\;\text{years}}{\text{Stiffness}\;\text{at}\;\text{age}\;20\;\text{years}}$$

The stiffening ratio has been formulated in this say to give an indication of the change in stiffness that occurs during the decades when the accommodation mechanism is in steady decline. From the curves specified in Heys et al. (2004) the values of SR (based on stiffness index) are 63 and 7.9 for the nucleus and cortex respectively. The equivalent data from the Model D age-stiffness model (based on shear modulus) are 59 and 3. This demonstrates that our data and the data of Heys et al. (2004) stiffen at a similar rate (although the agreement is closer for the nucleus than the cortex).

Fig. 1 in Heys et al. (2007) provides additional stiffness index data on the nucleus. These data (obtained using fresh lenses) are substantially greater in magnitude than the nucleus measurements in Heys et al. (2004). To compare them with our own shear modulus data an exponential function has been fitted to the stiffness index data plotted in Fig. 1 of Heys et al. (2007). The SR value obtained from this exponential function is 10. This value of stiffening ratio is considerably lower than the value deduced from Heys et al. (2004) and also from our own data.

### 6.3 Comparison with Weeber et al. (2007)

Weeber et al. (2007) describe a dynamic indentation method to measure lens stiffness. This test has certain similarities with the Heys et al. (2004) test (i.e. the lenses were frozen prior to testing, they were sectioned on an equatorial plane and a series of micro-indentation tests were conducted to determine the spatial variation of stiffness). The tests differ, however, in the important respect that in the Weeber et al. (2007) tests the probe was first penetrated a distance of 0.5 mm into the lens. The probe was then subjected to displacement cycles with amplitudes in the range 1–50 μm. The measured forces was then used (via a finite element-based calibration process) to determine values of complex shear modulus for small-amplitude cycles.

The magnitude of the oscillatory strain field developed around the probe is likely to be sufficiently small for the test to be capable of providing data on shear modulus that can reasonably be compared with the data from the current experiment (although it is noted that the cyclic indentation data may be affected by the unavoidable damage to the tissue that is caused by the initial 0.5 mm static penetration). It is noted that only 10 lenses were investigated in the study. It is also clear that the results given by Weeber et al. (2007) do not provide an entirely consistent pattern (for example the shear modulus profile for the 40-year lens in Fig. 3 of Weeber et al. (2007) suggests that the cortex of the lens is substantially stiffer than the older, 49-year, lens plotted in Fig. 4 of Weeber et al. (2007). Scatter of this sort may, of course, reveal genuine differences in the rates at which the lenses of two individuals stiffen with age. However, these variations have the consequence that the 10 lenses tested by Weeber et al. 2007 are probably too small in number for the purpose of specifying a complete model of the way in which the stiffness of the lens develops with age. Weeber et al. (2007) address this issue by describing the mathematical function that they fit to their data as an ‘exploratory model’.

The lenses tested by Weeber et al. (2007) were sectioned on the equatorial plane. At each test point the probe was inserted 0.5 mm into the lens substance before performing the oscillatory test; the measured shear modulus value presumably, therefore, corresponds to a location that is approximately a distance of 0.5 mm posterior of the equatorial plane. For the current comparison exercise it is assumed, for convenience, that the Weeber et al. (2007) measurements correspond to points located on the plane through the midpoint of the lens (which is assumed to lie 0.4 mm posterior to the equatorial plane for an isolated lens). For Model D the transition from the nucleus to the cortex is calculated to occur at a radius of 3.10 mm in this plane. The relative position for a given radius, *r*, in this plane is taken to be.

(14) $$p_{\text{r}}=\frac{2r}{D}$$

where *D* is the lens equatorial diameter determined from the relationship given by Rosen et al. (2006) as:

(15) D=8.7+0.0138×A

where *D* is in units of mm and *A* is age in units of years.

**Fig. 12:**
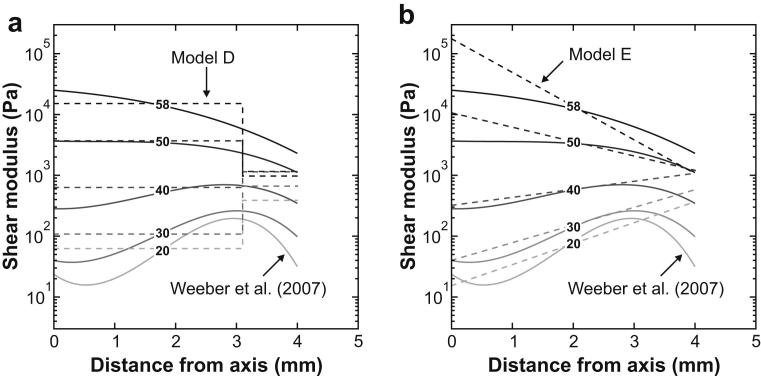
Comparison between the stiffness profiles reported by Weeber et al. (2007) (solid lines) and the age-stiffness models proposed in the current paper. Figure (a) gives a comparison between Model D (dashed lines) and the Weeber et al. (2007) data. Figure (b) gives a comparison between Model E (dashed lines) and the Weeber et al. (2007) data.

The current data are compared with the Weeber ‘exploratory’ model in Fig. 12. (Note that the precise form of the exploratory model is not specified by Weeber et al. (2007). The exploratory model plotted in Fig. 12 has therefore obtained by the authors from a separate curve fitting exercise on the exploratory model curves plotted in Weeber et al., 2007). Within the limitations of the respective representations, the stiffness profiles reported by Weeber et al. (2007) and the current age-stiffness models appear broadly similar in Fig. 12, at least for the inner region of the lens.

In the interior of younger lenses, Model E provides closer agreement with the profiles of Weeber et al. (2007) than Model D. If the stiffness of the inner region of the lens is well represented by the indentation profiles, then the capacity of Model E to more closely match that form provides an explanation for its better performance at matching the experimental results of the spinning lens test for lenses younger than 30 years (see Fig. 7). In the interior of the older lenses, Model E departs more dramatically from the indentation profiles than Model D. In the outer region of the lens the difference between the indentation profiles and Model E is similar to the difference seen in the cortex for Model D, with the results from the spinning test showing considerably smaller variation with age than the indentation data, especially for older lenses.

### 6.4 Comparison with the Hollman et al. (2007) data

**Fig. 13:**
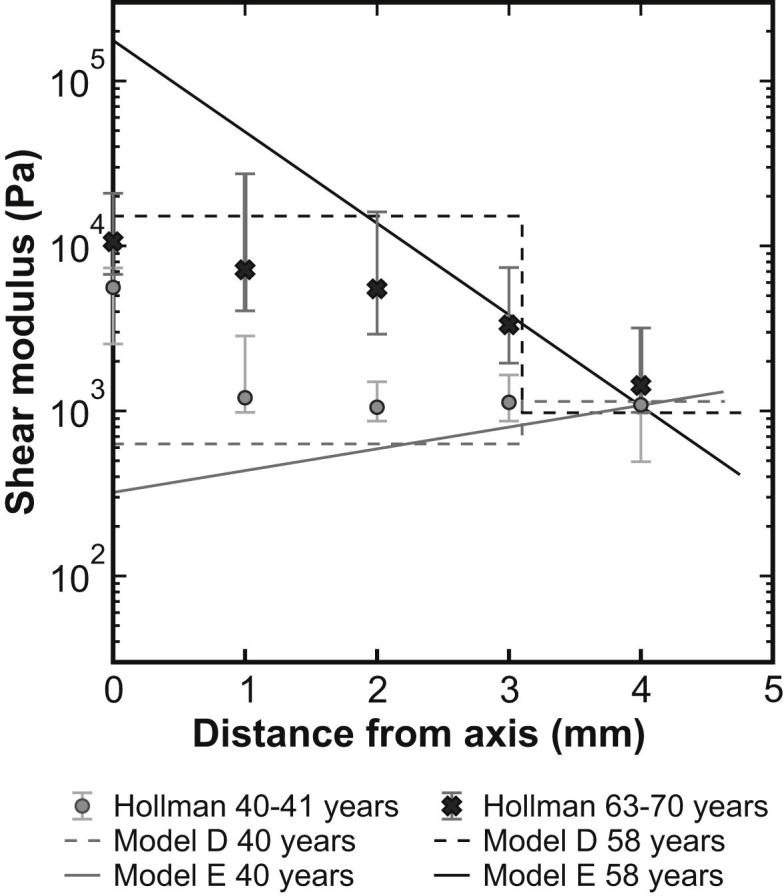
Comparison between the data of Hollman et al. (2007) and the current age-stiffness models for Models D and E. The error bars plotted are those given by Hollman et al. (2007).

Hollman et al. (2007) provide data on Young’s modulus at certain points within the lens, for lenses ages 40–41 years and 63–70 years, obtained by an acoustic method. These data are compared with the age-stiffness models derived from models D and E for ages 40 and 58 years in Fig. 13. (Note that the oldest lens tested in the current program is of age 58. The closest comparison that can be made, without resorting to extrapolation, between the older lenses tested by Hollman et al. (2007) and our own data is to use age 58 years in our age-stiffness model).

Some differences are obvious in the two sets of data. The Hollman et al. (2007) data for the 40–41 year lenses indicate that the central portion of the lens is stiffer than the peripheral regions. This contrasts with the current spinning lens data which indicates the reverse. The lenses of ages 63–70 years fall outside of the age range tested in the current study. It is therefore surprising to see that the Hollman stiffness data for the central portions of these lenses is rather less than the values for a 58-year lens as determined from the current age-stiffness models. Both the current data and those of Hollman et al. (2007) suggest a relatively small change in stiffness with age at a distance from the polar axis of 4 mm at the ages tested. This contrasts with the exploratory model of Weeber et al. (2007).

### 6.5 Conclusions

A set of stiffness data on 29 lenses in the age range 12 years to 58 years has been used to derive age-stiffness models for three forms of SVF. It is suggested that the two non-homogeneous models (Model D and Model E) have potential applications in the development of improved models of the accommodation process. Model H is regarded as being of less importance and is included in the current paper for the principal purpose of comparison with the other two models.

An important feature of our results (which is also evident in the previous data of Fisher (1971); Heys et al. (2004); Weeber et al., 2007) is that for young lenses the central regions of the lens are less stiff than the peripheral regions. This pattern is reversed in the older lenses. At some intermediate age the lens is approximately homogeneous in stiffness.

Detailed investigation of the reliability of the spinning lens shear modulus data appears to suggest that Model E may be preferable for the purposes of modelling young lenses (i.e. lenses aged less than about 30 years) and Model D may be preferable for older lenses (i.e. age greater than about 30 years).

An attempt has been made to compare the current data with comparable data in previous publications. Comparisons of this sort are not straightforward and few firm conclusions can be drawn. However, the outcome of this comparative study demonstrates that the spinning lens data presented in this paper lie within the broad spread of data determined by other researchers.

A key feature of our data is that they were obtained by making mechanical measurements on the entire lens substance. This approach naturally leads to models for spatial non-homogeneity that are in a form that can be readily incorporated within a finite element model of the accommodation process. It is hoped, therefore, that they will provide a worthwhile contribution to future studies on the mechanics of accommodation.

## Figures and Tables

**Table 1:** Primary test sequence.

Test reference	*A*_ref_	*A*	*B*_ref_	*B*	*C*_ref_
Rotational speed (rpm)	70	700	70	1000	70

**Table 2:** Shear modulus parameters for the SVF curves plotted in Fig. 6. Shear modulus data are in units of kPa.

Lens age	Model H	Model D	Model E
33 years	*G* = 0.475	*G*_N_ = 0.192; *G*_C_ = 0.933	*G*_0_ = 0.0619; *G*_1_ = 1.27
43 years	*G* = 1.33	*G*_N_ = 1.28; *G*_C_ = 1.41	*G*_0_ = 1.23; *G*_1_ = 1.40
50 years	*G* = 3.57	*G*_N_ = 7.48; *G*_C_ = 1.07	*G*_0_ = 59.6; *G*_1_ = 0.554

**Table 3:** Parameters for age-stiffness models. These parameters are determined to provide a best fit to the data obtained from Test B when conducted on the lenses in Set $\bar{G}$.

Model	Shear modulus parameter (Pa)	*A*^∗^ (years)	*b*_1_ (years^−1^)	*b*_2_ (years^−1^)	*c* (Pa)
H	*G*	23.8	0.00322	0.0424	175

D	*G*_N_	27.5	0.00562	0.0767	68.8
	*G*_C_	43.0	0.0235	−0.00932	1340.0

E	*G*_0_	35.6	0.0415	0.152	68.5
	*G*_1_	43.2	0.0191	−0.0380	1500.0

**Table 4:** The stiffness parameters for Model D calculated for primary and secondary tests on each of the representative lenses. Tests at 1000 rpm were used for the 33-year and 43-year lenses, while tests at 1400 rpm were used for the 50-year lens. The time data indicate the elapsed time between commencing the test sequence and collecting the relevant images.

Lens age	Primary test			Secondary test		
	*G*_N_ (kPa)	*G*_C_ (kPa)	Time (min)	*G*_N_ (kPa)	*G*_C_ (kPa)	Time (min)
33 years	0.19	0.93	8	0.21	1.00	19
43 years	1.28	1.41	8	1.27	1.57	20
50 years	7.35	1.16	9	7.06	1.27	18

## Footnotes

1 Other forms of SVF are possible. In the early stages of the project, for example, modelling was conducted using a linear variation of shear modulus with relative position. This particular approach, however, was found to suffer from the difficulty that the optimisation process can lead to zero values of shear modulus at *p*_r_ = 0 or *p*_r_ = 1. This is clearly unrealistic. A linear SVF was therefore not included in the current study.
