# Supplementary material for: Shear modulus data for the human lens determined from a spinning lens test
Source: Exp Eye Res. 2012 Apr;97(1):36–48. doi: 10.1016/j.exer.2012.01.011 (PMC3405528; doi:10.1016/j.exer.2012.01.011)
Supplement: Supplementary file 1 [file mmc1.pdf]

Description of Supplementary Material

25th February 2012

A wide range of details on the specification of the lenses that were tested in the study described in this paper, and also the inferred values of shear modulus, are given in Wilde (2011). A selection of these details are provided here as supplementary material. It is hoped that the data in these tables are reasonably self-explanatory. For further information see Wilde (2011).

Reference

Wilde, G.S., 2011. Measurement of human lens stiffness for modelling presbyopia treatments. D.Phil. Thesis, University of Oxford, UK.
