# Supplementary material for: Shear modulus data for the human lens determined from a spinning lens test
Source: Exp Eye Res. 2012 Apr;97(1):36–48. doi: 10.1016/j.exer.2012.01.011 (PMC3405528; doi:10.1016/j.exer.2012.01.011)
Supplement: Supplementary file 2 [file mmc2.pdf]

Table S.1 – *Lenses received during preliminary tests.*

**Oxford label**	**sex**	**age** (years)
L001A	M	23
L001B	M	23
L002A	M	35
L002B	M	35
L003A	F	74
L003B	F	74
L004A	F	56
L004B	F	56
L005A	F	64
L005B	F	64
L006A	M	65
L006B	M	65
L007A	F	17
L007B	F	17
L008A	M	43
L008B	M	43
L009A	F	59
L009B	F	59
L010A	M	61

**Oxford label**	**sex**	**age** (years)
L011A	M	87
L011B	M	87
L012A	M	52
L012B	M	52
L013A	M	76
L013B	M	76
L014A	M	66
L014B	M	66
L015A	M	68
L015B	M	68
L016A	M	22
L016B	M	22
L017A	M	53
L017B	M	53
L018A	M	47
L018B	M	47
L019A	M	54
L019B	M	54

Generated on the 25th of February 2012.

Table S.2 – *The lenses received for the main spinning lens tests. Aspect ratios were not calculated for lenses L020B, L025B, and L058B because they were damaged or had absent capsules when received. The table continues over four pages.*

**Oxford label**	**sex**	**age** (years)	**time since death** (days)	**aspect ratio**, α	**set** $\mathcal{G}$	**swelling**	**damage**	**fluid**
L020A	F	25	*not tested due to absence of capsule*					
L020B	F	25	2 22:25	–			•	
L021A	F	40	2 14:42	2.324	•			
L021B	F	40	2 16:12	2.289				•
L022A	M	26	2 14:20	1.853		•		•
L022B	M	26	2 15:01	1.950	•			
L023A	M	49	1 22:09	2.756			•	
L023B	M	49	*not tested due to damage*					
L024A	M	63	2 03:35	1.819		•	•	
L024B	M	63	2 04:42	1.879		•		
L025A	M	37	*not tested due to absence of capsule*					
L025B	M	37	3 09:33	–			•	
L026A	F	30	1 12:38	1.895		•		
L026B	F	30	1 13:37	1.880		•		•
L027A	M	46	2 15:14	2.264	•			
L027B	M	46	3 21:01	2.271	•			
L028A	M	31	2 15:10	2.325			•	
L028B	M	31	2 18:00	2.212			•	
L029A	M	49	2 17:19	2.206	•			
L029B	M	49	3 19:08	2.184	•			

Table S.2 – *(Part ii, continued from previous page.)*

**Oxford label**	**sex**	**age** (years)	**time since death** (days)	**aspect ratio**, α	**set** $\mathcal{G}$	**swelling**	**damage**	**fluid**
L030A	M	58	2 12:20	2.306				•
L030B	M	58	2 13:36	2.264	•			
L031A	M	21	2 16:31	2.152			•	•
L031B	M	21	2 19:25	2.122				•
L032A	F	45	2 13:49	1.514		•	•	
L032B	F	45	2 15:06	1.910		•	•	
L033A	F	19	2 09:08	1.794	•			
L033B	F	19	2 10:07	1.846				•
L034A	M	43	*not tested due to absence of author*					
L034B	M	43	"					
L035A	M	65	"					
L035B	M	65	"					
L036A	M	53	"					
L036B	M	53	"					
L037A	M	12	1 22:40	1.944	•			
L037B	M	12	1 23:40	1.949				•
L038A	F	33	4 14:11	2.246	•			
L038B	F	33	4 17:11	2.256				•
L039A	M	43	4 03:55	2.167			•	
L039B	M	43	4 04:55	2.255	•			

Table S.2 – *(Part iii, continued from previous page.)*

**Oxford label**	**sex**	**age** (years)	**time since death** (days)	**aspect ratio**, α	**set** $\mathcal{G}$	**swelling**	**damage**	**fluid**
L040A	M	23	4 00:43	1.980	•			
L040B	M	23	4 01:38	1.926	•			
L041A	M	39	3 01:21	1.913		•	•	
L041B	M	39	3 02:21	2.003			•	
L042A	M	40	2 18:27	1.905		•		
L042B	M	40	2 19:42	1.917		•		
L043A	M	12	2 16:05	1.828	•			
L043B	M	12	2 16:55	1.741	•			
L044A	F	44	3 14:38	2.111			•	
L044B	F	44	3 16:08	2.156	•			
L045A	M	45	3 04:20	1.870		•		•
L045B	M	45	3 07:45	1.828		•	•	
L046A	F	58	3 06:48	1.923		•	•	
L046B	F	58	3 08:38	1.896		•	•	
L047A	M	43	2 16:55	2.322				•
L047B	M	43	2 20:20	2.337	•			
L048A	M	48	2 21:43	2.046				•
L048B	M	48	3 00:51	2.157				•
L049A	M	60	2 07:53	1.829		•		•
L049B	M	60	2 09:15	1.821		•	•	

Table S.2 – *(Part iv, continued from previous page.)*

**Oxford label**	**sex**	**age** (years)	**time since death** (days)	**aspect ratio**, α	**set** $\mathcal{G}$	**swelling**	**damage**	**fluid**
L050A	F	48	2 08:27	2.178	•			
L050B	F	48	2 09:23	2.176	•			
L051A	M	40	3 02:36	2.057	•			
L051B	M	40	3 03:41	1.934		•		•
L052A	F	34	2 18:20	1.873		•		
L052B	F	34	2 21:23	2.260	•			
L053A	F	56	2 17:13	2.055	•			
L053B	F	56	3 09:41	1.988			•	
L054A	M	52	3 12:12	2.006	•			
L054B	M	52	4 01:19	1.995	•			
L055A	M	51	3 14:35	2.137	•			
L055B	M	51	2 23:23	2.107	•			
L056A	M	50	2 14:02	2.323	•			
L056B	M	50	2 16:16	2.353	•			
L057A	M	58	2 04:46	2.011				•
L057B	M	58	2 06:28	2.081	•			
L058A	M	63	1 19:47	1.816		•		
L058B	M	63	2 21:12	–			•	
L059A	F	41	2 07:12	1.913		•		
L059B	F	41	3 06:25	1.938		•		
