# Supplementary material for: Shear modulus data for the human lens determined from a spinning lens test
Source: Exp Eye Res. 2012 Apr;97(1):36–48. doi: 10.1016/j.exer.2012.01.011 (PMC3405528; doi:10.1016/j.exer.2012.01.011)
Supplement: Supplementary file 3 [file mmc3.pdf]

Table S.3 – *Stiffness parameters for lenses of set $\mathcal{G}$ calculated from test* B *(1000 rpm) using support constraint F. If this is the preferred constraint for a given lens it is marked in that column.*

	**model H**	**model D**		**model E**		**preferred**
	G (Pa)	G_N (Pa)	G_C (Pa)	G_0 (Pa)	G_1 (Pa)	**constraint**
L021A	7.52×10^{2}	4.11×10^{2}	1.21×10^{3}	1.71×10^{2}	1.51×10^{3}	
L022B	1.80×10^{2}	7.40×10^{1}	4.15×10^{2}	3.02×10^{1}	4.45×10^{2}	•
L027A	1.06×10^{3}	7.54×10^{2}	1.47×10^{3}	4.34×10^{2}	1.65×10^{3}	
L027B	9.04×10^{2}	7.46×10^{2}	1.17×10^{3}	4.72×10^{2}	1.34×10^{3}	
L029A	2.06×10^{3}	3.30×10^{3}	9.77×10^{2}	1.22×10^{4}	6.21×10^{2}	
L029B	2.28×10^{3}	3.07×10^{3}	1.53×10^{3}	5.69×10^{3}	1.31×10^{3}	
L030B	3.30×10^{3}	8.41×10^{3}	1.07×10^{3}	1.34×10^{5}	3.30×10^{2}	
L033A	1.49×10^{2}	5.88×10^{1}	2.70×10^{2}	1.42×10^{1}	3.67×10^{2}	•
L037A	1.80×10^{2}	6.44×10^{1}	3.50×10^{2}	7.41×10^{0}	5.38×10^{2}	•
L038A	4.37×10^{2}	8.89×10^{1}	9.77×10^{2}	1.97×10^{1}	1.55×10^{3}	
L039B	1.22×10^{3}	1.09×10^{3}	1.42×10^{3}	8.81×10^{2}	1.52×10^{3}	
L040A	1.76×10^{2}	7.05×10^{1}	3.86×10^{2}	1.60×10^{1}	5.16×10^{2}	•
L040B	1.85×10^{2}	5.50×10^{1}	3.90×10^{2}	8.79×10^{0}	5.91×10^{2}	•
L043A	1.62×10^{2}	6.43×10^{1}	2.68×10^{2}	9.93×10^{0}	4.26×10^{2}	•
L043B	1.46×10^{2}	4.52×10^{1}	2.82×10^{2}	8.26×10^{0}	4.17×10^{2}	•
L044B	8.72×10^{2}	4.14×10^{2}	1.66×10^{3}	1.45×10^{2}	2.13×10^{3}	
L047B	1.25×10^{3}	7.83×10^{2}	1.97×10^{3}	3.87×10^{2}	2.33×10^{3}	
L050A	2.26×10^{3}	2.73×10^{3}	1.74×10^{3}	3.72×10^{3}	1.63×10^{3}	
L050B	2.19×10^{3}	2.69×10^{3}	1.58×10^{3}	3.79×10^{3}	1.51×10^{3}	
L051A	6.71×10^{2}	3.77×10^{2}	1.15×10^{3}	1.68×10^{2}	1.35×10^{3}	
L052B	4.52×10^{2}	1.14×10^{2}	9.87×10^{2}	2.76×10^{1}	1.52×10^{3}	
L053A	3.02×10^{3}	1.38×10^{4}	8.61×10^{2}	1.55×10^{5}	3.36×10^{2}	
L054A	2.81×10^{3}	3.63×10^{3}	1.90×10^{3}	5.47×10^{3}	1.73×10^{3}	
L054B	2.71×10^{3}	4.71×10^{3}	1.48×10^{3}	1.02×10^{4}	1.21×10^{3}	
L055A	1.98×10^{3}	3.59×10^{3}	8.47×10^{2}	1.13×10^{4}	6.28×10^{2}	
L055B	1.94×10^{3}	3.58×10^{3}	8.99×10^{2}	1.11×10^{4}	6.82×10^{2}	
L056A	3.31×10^{3}	6.03×10^{3}	1.19×10^{3}	3.61×10^{4}	6.97×10^{2}	
L056B	3.19×10^{3}	6.60×10^{3}	1.06×10^{3}	3.90×10^{4}	6.42×10^{2}	
L057B	3.64×10^{3}	7.29×10^{3}	1.16×10^{3}	3.90×10^{4}	7.43×10^{2}	

Generated on the 25th of February 2012.

***Table S.4** – Stiffness parameters calculated for some lenses of set $\mathcal{G}$ from test* A *(700 rpm) using support constraint F. If this is the preferred constraint for a given lens it is marked in that column.*

	**model H**	**model D**		**model E**		**preferred**
	G (Pa)	G_N (Pa)	G_C (Pa)	G_0 (Pa)	G_1 (Pa)	**constraint**
L022B	1.54×10^2	5.42×10^1	2.93×10^2	6.08×10^0	5.77×10^2	•
L033A	1.31×10^2	5.24×10^1	2.45×10^2	1.13×10^1	3.54×10^2	•
L037A	1.45×10^2	4.39×10^1	2.81×10^2	5.04×10^0	4.12×10^2	•
L038A	2.97×10^2	8.79×10^1	7.99×10^2	2.54×10^1	1.09×10^3	
L040A	1.69×10^2	5.68×10^1	3.36×10^2	7.56×10^0	5.37×10^2	•
L040B	1.66×10^2	4.78×10^1	3.19×10^2	6.34×10^0	5.08×10^2	•
L043A	1.28×10^2	3.93×10^1	2.55×10^2	9.30×10^0	3.19×10^2	•
L043B	1.19×10^2	4.21×10^1	2.33×10^2	8.89×10^0	3.32×10^2	•
L052B	2.92×10^2	5.78×10^0	1.22×10^3	1.90×10^1	1.18×10^3	

***Table S.5** – Stiffness parameters calculated for some lenses of set $\mathcal{G}$ from test* C *(1400 rpm) using support constraint F. If this is the preferred constraint for a given lens it is marked in that column.*

	**model H**	**model D**		**model E**		**preferred**
	G (Pa)	G_N (Pa)	G_C (Pa)	G_0 (Pa)	G_1 (Pa)	**constraint**
L029A	2.17×10^3	2.96×10^3	1.30×10^3	8.79×10^3	8.92×10^2	
L029B	2.21×10^3	2.90×10^3	1.44×10^3	5.09×10^3	1.27×10^3	
L030B	3.95×10^3	7.14×10^3	1.35×10^3	5.76×10^5	2.20×10^2	
L047B	1.27×10^3	7.46×10^2	2.13×10^3	3.17×10^2	2.65×10^3	
L050A	2.41×10^3	2.99×10^3	1.64×10^3	4.47×10^3	1.55×10^3	
L050B	2.33×10^3	2.67×10^3	1.86×10^3	3.30×10^3	1.83×10^3	
L051A	7.67×10^2	4.09×10^2	1.38×10^3	1.62×10^2	1.70×10^3	
L053A	2.96×10^3	1.35×10^4	8.56×10^2	1.53×10^5	3.31×10^2	
L054A	2.71×10^3	4.33×10^3	1.44×10^3	9.77×10^3	1.15×10^3	
L054B	2.88×10^3	5.68×10^3	1.45×10^3	1.35×10^4	1.16×10^3	
L055A	2.10×10^3	3.92×10^3	8.86×10^2	1.33×10^4	6.38×10^2	
L055B	2.12×10^3	3.98×10^3	9.36×10^2	1.29×10^4	6.99×10^2	
L056A	3.48×10^3	6.02×10^3	1.15×10^3	3.49×10^4	7.18×10^2	
L056B	3.36×10^3	6.32×10^3	1.18×10^3	3.33×10^4	7.58×10^2	
L057B	3.67×10^3	7.55×10^3	1.20×10^3	4.98×10^4	6.86×10^2	

Table S.6 – *Stiffness parameters for lenses of set $\mathcal{G}$ calculated from test* B *(1000 rpm) using support constraint S. If this is the preferred constraint for a given lens it is marked in that column.*

	model H	model D		model E		preferred
	G (Pa)	G_N (Pa)	G_C (Pa)	G_0 (Pa)	G_1 (Pa)	constraint
L021A	8.11×10^{2}	6.53×10^{2}	1.06×10^{3}	4.65×10^{2}	1.15×10^{3}	•
L022B	2.38×10^{2}	2.02×10^{2}	3.27×10^{2}	1.80×10^{2}	2.98×10^{2}	
L027A	1.13×10^{3}	1.08×10^{3}	1.26×10^{3}	9.77×10^{2}	1.28×10^{3}	•
L027B	1.01×10^{3}	9.50×10^{2}	1.12×10^{3}	8.72×10^{2}	1.11×10^{3}	•
L029A	2.17×10^{3}	4.02×10^{3}	8.98×10^{2}	1.74×10^{4}	5.53×10^{2}	•
L029B	2.50×10^{3}	3.70×10^{3}	1.37×10^{3}	8.84×10^{3}	1.09×10^{3}	•
L030B	3.55×10^{3}	9.59×10^{3}	1.03×10^{3}	1.41×10^{5}	3.30×10^{2}	•
L033A	1.80×10^{2}	2.02×10^{2}	1.57×10^{2}	3.23×10^{2}	1.38×10^{2}	
L037A	2.21×10^{2}	2.11×10^{2}	2.36×10^{2}	2.79×10^{2}	1.95×10^{2}	
L038A	4.75×10^{2}	1.92×10^{2}	9.33×10^{2}	6.19×10^{1}	1.27×10^{3}	•
L039B	1.33×10^{3}	1.28×10^{3}	1.41×10^{3}	1.23×10^{3}	1.40×10^{3}	•
L040A	2.41×10^{2}	1.91×10^{2}	3.16×10^{2}	1.67×10^{2}	2.92×10^{2}	
L040B	2.30×10^{2}	1.96×10^{2}	2.72×10^{2}	2.19×10^{2}	2.54×10^{2}	
L043A	1.87×10^{2}	2.16×10^{2}	1.57×10^{2}	3.70×10^{2}	1.36×10^{2}	
L043B	1.79×10^{2}	2.18×10^{2}	1.36×10^{2}	3.63×10^{2}	1.28×10^{2}	
L044B	9.45×10^{2}	7.34×10^{2}	1.40×10^{3}	5.13×10^{2}	1.46×10^{3}	•
L047B	1.34×10^{3}	1.10×10^{3}	1.76×10^{3}	7.13×10^{2}	1.99×10^{3}	•
L050A	2.55×10^{3}	3.34×10^{3}	1.53×10^{3}	5.87×10^{3}	1.36×10^{3}	•
L050B	2.36×10^{3}	3.25×10^{3}	1.38×10^{3}	6.05×10^{3}	1.22×10^{3}	•
L051A	7.14×10^{2}	5.13×10^{2}	1.11×10^{3}	3.01×10^{2}	1.20×10^{3}	•
L052B	4.81×10^{2}	2.29×10^{2}	9.30×10^{2}	8.28×10^{1}	1.23×10^{3}	•
L053A	3.50×10^{3}	1.84×10^{4}	8.30×10^{2}	2.01×10^{5}	3.13×10^{2}	•
L054A	3.02×10^{3}	4.45×10^{3}	1.63×10^{3}	8.41×10^{3}	1.42×10^{3}	•
L054B	2.89×10^{3}	6.04×10^{3}	1.29×10^{3}	1.57×10^{4}	9.97×10^{2}	•
L055A	2.14×10^{3}	4.27×10^{3}	7.96×10^{2}	1.48×10^{4}	5.74×10^{2}	•
L055B	2.08×10^{3}	4.06×10^{3}	8.60×10^{2}	1.56×10^{4}	5.95×10^{2}	•
L056A	3.61×10^{3}	7.23×10^{3}	1.10×10^{3}	5.31×10^{4}	6.15×10^{2}	•
L056B	3.57×10^{3}	7.48×10^{3}	1.07×10^{3}	5.96×10^{4}	5.54×10^{2}	•
L057B	3.98×10^{3}	8.19×10^{3}	1.12×10^{3}	5.16×10^{4}	6.76×10^{2}	•

Table S.7 – *Stiffness parameters calculated for some lenses of set $\mathcal{G}$ from test* A *(700 rpm) using support constraint S. If this is the preferred constraint for a given lens it is marked in that column.*

	model H	model D		model E		preferred
	G (Pa)	G_N (Pa)	G_C (Pa)	G_0 (Pa)	G_1 (Pa)	constraint
L022B	2.11×10^{2}	1.69×10^{2}	2.72×10^{2}	2.06×10^{2}	2.15×10^{2}	
L033A	1.56×10^{2}	1.92×10^{2}	1.13×10^{2}	3.38×10^{2}	1.07×10^{2}	
L037A	1.60×10^{2}	1.61×10^{2}	1.63×10^{2}	3.75×10^{2}	1.08×10^{2}	
L038A	3.47×10^{2}	1.77×10^{2}	8.27×10^{2}	9.13×10^{0}	1.93×10^{3}	•
L040A	1.98×10^{2}	1.56×10^{2}	2.63×10^{2}	1.47×10^{2}	2.27×10^{2}	
L040B	1.90×10^{2}	1.65×10^{2}	2.18×10^{2}	2.41×10^{2}	1.65×10^{2}	
L043A	1.41×10^{2}	1.77×10^{2}	1.06×10^{2}	5.00×10^{2}	7.96×10^{1}	
L043B	1.41×10^{2}	2.30×10^{2}	6.50×10^{1}	3.80×10^{2}	8.88×10^{1}	
L052B	3.53×10^{2}	2.12×10^{2}	8.32×10^{2}	4.68×10^{1}	1.36×10^{3}	•

Table S.8 – *Stiffness parameters calculated for some lenses of set $\mathcal{G}$ from test* C *(1400 rpm) using support constraint S. If this is the preferred constraint for a given lens it is marked in that column.*

	model H	model D		model E		preferred
	G (Pa)	G_N (Pa)	G_C (Pa)	G_0 (Pa)	G_1 (Pa)	constraint
L029A	2.38×10^{3}	3.39×10^{3}	1.19×10^{3}	1.51×10^{4}	7.03×10^{2}	•
L029B	2.39×10^{3}	3.58×10^{3}	1.26×10^{3}	8.06×10^{3}	1.04×10^{3}	•
L030B	4.35×10^{3}	8.26×10^{3}	1.29×10^{3}	1.03×10^{6}	1.81×10^{2}	•
L047B	1.35×10^{3}	9.35×10^{2}	2.21×10^{3}	5.01×10^{2}	2.57×10^{3}	•
L050A	2.65×10^{3}	3.56×10^{3}	1.46×10^{3}	6.68×10^{3}	1.31×10^{3}	•
L050B	2.55×10^{3}	3.26×10^{3}	1.61×10^{3}	5.32×10^{3}	1.49×10^{3}	•
L051A	8.23×10^{2}	5.36×10^{2}	1.43×10^{3}	2.82×10^{2}	1.60×10^{3}	•
L053A	3.19×10^{3}	1.77×10^{4}	8.28×10^{2}	2.09×10^{5}	3.02×10^{2}	•
L054A	2.89×10^{3}	5.32×10^{3}	1.27×10^{3}	1.32×10^{4}	1.04×10^{3}	•
L054B	3.10×10^{3}	7.00×10^{3}	1.33×10^{3}	1.90×10^{4}	1.01×10^{3}	•
L055A	2.28×10^{3}	4.62×10^{3}	8.38×10^{2}	1.72×10^{4}	5.86×10^{2}	•
L055B	2.32×10^{3}	4.50×10^{3}	9.06×10^{2}	1.69×10^{4}	6.38×10^{2}	•
L056A	3.86×10^{3}	6.41×10^{3}	1.14×10^{3}	5.80×10^{4}	5.92×10^{2}	•
L056B	3.74×10^{3}	7.35×10^{3}	1.16×10^{3}	4.99×10^{4}	6.64×10^{2}	•
L057B	4.04×10^{3}	8.84×10^{3}	1.14×10^{3}	7.73×10^{4}	5.88×10^{2}	•
